# Resveratrol Promotes Diabetic Wound Healing via SIRT1-FOXO1-c-Myc Signaling Pathway-Mediated Angiogenesis

**DOI:** 10.3389/fphar.2019.00421

**Published:** 2019-04-24

**Authors:** Xiaozhong Huang, Jia Sun, Gen Chen, Chao Niu, Ying Wang, Congcong Zhao, Jian Sun, Huiya Huang, Shuai Huang, Yangzhi Liang, Yingjie Shen, Weitao Cong, Litai Jin, Zhongxin Zhu

**Affiliations:** ^1^Department of Pediatric Surgery, The Second Affiliated Hospital and Yuying Children’s Hospital of Wenzhou Medical University, Wenzhou, China; ^2^School of Pharmaceutical Science, Wenzhou Medical University, Wenzhou, China; ^3^Pediatric Research Institute, The Second Affiliated Hospital and Yuying Children’s Hospital of Wenzhou Medical University, Wenzhou, China; ^4^Department of Pharmacy, Jinhua Women & Children Health Hospital, Jinhua, China; ^5^Department of Intensive Care Unit, The First Affiliated Hospital of Wenzhou Medical University, Wenzhou, China

**Keywords:** angiogenesis, diabetic wound healing, endothelial dysfunction, silent information regulator 1, forkhead box O1, c-Myc

## Abstract

**Background/Aims:** Diabetic non-healing skin ulcers represent a serious challenge in clinical practice, in which the hyperglycemia-induced disturbance of angiogenesis, and endothelial dysfunction play a crucial role. Resveratrol (RES), a silent information regulator 1 (SIRT1) agonist, can improve endothelial function and has strong pro-angiogenic properties, and has thus become a research focus for the treatment of diabetic non-healing skin ulcers; however, the underlying mechanism by which RES regulates these processes remains unclear. Therefore, the present study was intended to determine if RES exerts its observed protective role in diabetic wound healing by alleviating hyperglycemia-induced endothelial dysfunction and the disturbance of angiogenesis.

**Methods:** We investigated the effects of RES on cell migration, cell proliferation, apoptosis, tube formation, and the underlying molecular mechanisms in 33 mM high glucose-stimulated human umbilical vein endothelial cells (HUVECs) by semi-quantitative RT-PCR, western blot analysis, terminal deoxynucleotidyl transferase-mediated dUTP nick end labeling (TUNEL) staining, and immunofluorescence *in vitro*. We further explored the role of RES on endothelial dysfunction and wound healing disturbance in db/db mice by TUNEL staining, immunofluorescence, and photography *in vivo*.

**Results:** We observed an obvious inhibition of hyperglycemia-triggered endothelial dysfunction and a disturbance of angiogenesis, followed by the promotion of diabetic wound healing via RES, along with restoration of the activity of the hyperglycemia-impaired SIRT1 signaling pathway. Pretreatment with EX-527, a SIRT1 inhibitor, abolished the RES-mediated endothelial protection and pro-angiogenesis action, and then delayed diabetic wound healing. Furthermore, examination of the overexpression of forkhead box O1 (FOXO1), a transcription factor substrate of SIRT1, in HUVECs and db/db mice revealed that RES activated SIRT1 to restore hyperglycemia-triggered endothelial dysfunction and disturbance of angiogenesis, followed by the promotion of diabetic wound healing in a c-Myc-dependent manner. Pretreatment with 10058-F4, a c-Myc inhibitor, repressed RES-mediated endothelial protection, angiogenesis, and diabetic wound healing.

**Conclusion:** Our findings indicate that the positive role of RES in diabetic wound healing via its SIRT1-dependent endothelial protection and pro-angiogenic effects involves the inhibition of FOXO1 and the de-repression of c-Myc expression.

## Introduction

Diabetes mellitus is a metabolic disease with an increasing incidence worldwide (Zimmet et al., 2014). The disease often leads to the development of serious complications such as microangiopathy, mainly including retinopathy, nephropathy, neuropathy, and diabetic non-healing skin ulcers (Zheng et al., 2018). Diabetic non-healing skin ulcers such as foot ulcers are caused by diminished wound healing and are among the most serious and costly complications associated with diabetes mellitus. Approximately 20% of moderate or severe diabetic foot ulcers lead to some level of amputation (Lavery et al., 2003; Lipsky et al., 2012).

Angiogenesis, the growth of new blood vessels or neovascularization to nourish damaged tissues, is critical to wound healing, and its disruption plays a major role in the formation of diabetic non-healing skin ulcers (Ackermann et al., 2014). Thus, a central aim of diabetic non-healing skin ulcers therapy is to improve angiogenesis. Vascular endothelial cells have key roles in angiogenesis and the wound healing process (Sawada et al., 2014). However, endothelial dysfunction is the earliest and most fundamental pathological change in diabetes and is responsible for many cardiovascular complications (Wils et al., 2017). Current diabetic non-healing skin ulcers treatments have substantial side effects and are expensive, which results in frequent non-compliance (McLaughlin et al., 2017). Thus, more effective therapeutic methods are needed.

Resveratrol (RES) is a naturally occurring polyphenol and phytoalexin that is enriched in mulberries, *Polygonum cuspidatum*, grapes, and red wine (Jhaveri et al., 2018). RES has been identified as a possible agonist of silent information regulator 1 (SIRT1), which is a master regulator of energy homeostasis. Previous research has demonstrated that RES improves endothelial function and exerts antidiabetic effects, but most of these studies have mainly focused on its antioxidant functions (Eroğlu et al., 2015; Gokce et al., 2017; Wu et al., 2018). Because clinical trials have revealed the ineffectiveness of antioxidants commonly employed to treat diabetic complications, it is possible that other important factors are involved in the development of these complications.

Beside its well-known anti-oxidant activity (de la Lastra and Villegas, 2007), RES exerts many biological effects mediated by SIRT1, a member of the NAD^+^-dependent Sir2 histone deacetylase family (Bashmakov et al., 2011). SIRT1 has been shown to regulate the expression of several genes that are involved in vascular endothelial homeostasis and remodeling with high expression within vasculature undergoing blood vessel growth, where it controls the angiogenic activity of endothelial cells (Das et al., 2018). Accordingly, SIRT1 has demonstrable protective effects against endothelial dysfunction through the prevention of the stress response, which is considered a target for the treatment of human pathologies such as diabetic complications.

As an important downstream molecule of SIRT1, forkhead box O1 (FOXO1) is a critical checkpoint of endothelial growth, which can restrict vascular expansion (Wilhelm et al., 2016). Behl et al. found that high glucose induces apoptosis and loss of rat microvascular endothelial cells in type 1 and type 2 diabetic rats by activating FOXO1 (Behl et al., 2009). Furthermore, FOXO1 has been reported to be a negative regulator of c-Myc in endothelial cells (Wilhelm et al., 2016). c-Myc regulates glycolysis, proliferation and mitochondrial function of vascular endothelial cells, thus extensively participating in various endothelial biological processes, such as angiogenesis (Baudino et al., 2002).

This study aimed to investigate whether RES accelerates the diabetic wound healing via its SIRT1-dependent pro-angiogenic effect. Further research find that the positive role of RES in the diabetic wound healing involves inhibition of FOXO1 and further de-repression of c-Myc, thus the new role of RES in mediating diabetic wound healing is elaborated.

## Materials and Methods

### Animal Procedures

Diabetic mice and their control littermates (db/db and db/m, respectively), were obtained from Jackson Laboratory (Strain: BKS.Cg-Dock7^m+/+^Lepr^db/J^), while C57BL/6 mice were acquired from the Model Animal Research Center at Nanjing University. RES (Sigma-Aldrich, St. Louis, MO, United States, R5010) was administered at a 50 mg/kg/day dose to the 8-week-old mice for 4 weeks (Kanamori et al., 2015). The db/m and db/db control groups received phosphate-buffered saline (PBS; Gibco, 10010) on the same schedule as the control groups. After a 4-week treatment course, corresponding analyses were performed. For the signaling pathway analysis, the pathway antagonist EX-527 (Selleck, S1541), an inhibitor of SIRT1, was administered at a 5 mg/kg/day dose (Zhao et al., 2015). The c-Myc inhibitor 10058-F4 (Selleck, S7153) was administered at a 30 mg/kg/day dose (Wang et al., 2017). For 4 weeks, these reagents were administered via osmotic minipumps subcutaneously embedded within the mice (ALZET, 1002; DURECT, Cupertino, CA, United States).

A mouse strain was obtained with the entire *SIRT1* exon 4, which encodes the complete mature SIRT1 peptide and is flanked by two loxP sites. Additionally, transgenic *Tie2-Cre* mice with pan-endothelial Cre recombinase expression were obtained in order to conduct vascular endothelium-specific gene manipulation (Anggrahini et al., 2009). The two strains were bred to obtain mice with endothelial-specific disruption of *SIRT1*. For experimental purposes, 8-week-old *SIRT1* flox/flox; *Tie2-Cre* (+) mice and control *SIRT1* flox/flox; *Tie2-Cre* (-) littermates were used. These mice were kindly provided by Dr. Yuqiang Ding (Department of Anatomy and Neurobiology, Collaborative Innovation Center for Brain Science, Tongji University School of Medicine, China).

All animals were housed under controlled conditions (22 ± 2°C, 60 ± 5% relative humidity, 12-h light/dark cycle). All experimental methods employed in this research followed ethical animal research guidelines meeting the approval of the Institutional Animal Care and Use Committee of Wenzhou Medical University (wydw2017-0026).

### Cell Culture

Human umbilical vein endothelial cells (HUVECs) obtained from Lonza were cultured in endothelial cell growth medium-2 (EGM-2 BulletKit, Lonza, CC-3156 & CC-4176) before the experiment. Subconfluent cells obtained after five to seven passages were used in the following experiments. Twelve hours prior to the cell culture procedures, all stock media were removed and replaced with phenol red-free low-glucose D-MEM (Gibco, 11054020) supplemented with 1% calf serum (Gibco, 16010159). HUVECs were transferred to EGM-2 consisting of either high glucose (HG, 33 mM) or normal glucose (NG, 5.5 mM) with or without 10 μM RES (Li et al., 2011) for 72 h. Osmotic control of the HG treatment was achieved using mannitol (5.5 mM glucose + 27.5 mM _D_-mannitol = 33 mM). Every 24 h, the media were replaced. For the signaling pathway analysis, the pathway antagonists EX-527 (10 μM) (Selleck, S1541), 10058-F4 (50 μM) (Selleck, S7153), and MG-132 (0.5 μM) (Selleck, S2619) were pretreated for 2 h each day prior to RES administration.

### Aortic Ring Assays

To establish the direct action of RES on vasculature, the thoracic aortae of 8-week-old mice from each line were isolated surgically, thoroughly cleaned, and dissected into 0.5-mm rings, which were then embedded in 1 mg mL^-1^ type-I collagen (Millipore, 08-115) in a 96-well plate as previously described (Aplin et al., 2008; Baker et al., 2011). The embedded rings were cultured in NG or HG (5.5 mM or 33 mM, respectively) serum-free endothelial basal medium (EBM) (Lonza, CC-3121) with or without RES (10 μM). Here, too, osmotic control in the HG treatment was achieved using mannitol (5.5 mM glucose + 27.5 mM D-mannitol = 33 mM). During the exponential growth phase, angiogenic response data were obtained by counting the endothelial microvessel sprouts growing out from the cultured rings. Rings were fixed for CD31 (Abcam, ab24590) immunofluorescence staining prior to the regression phase. On day 12, images were captured, from which the total number of branches under each treatment were counted using ImageJ (National Institutes of Health, Bethesda, MD, United States).

### *In vitro* Angiogenesis (Tube Formation) Assay

Matrigel tube formation assays were used to assess the *in vitro* angiogenic activity of HUVECs. Following the completion of the aforementioned experimental protocol, calcein (Corning, 354216), a cell-permeable dye, was used to stain the HUVECs. After a 30-min incubation, the HUVECs were replated onto Matrigel-precoated 24-well plates (with 150 μL/well of growth factor–reduced Matrigel; Corning, 354234), which were transferred to a 37°C cell culture incubator for 12 h. After incubation, a computer-assisted microscope (EVOS, Thermo Fisher Scientific, MA, United States) was used to assess capillary-like tube formation, as defined by the presence of tube-like structures at least four times as long their widths. The tube lengths in duplicate wells were counted and averaged using ImageJ software.

### Immunoblotting Analysis

Briefly, 30-μg subsamples of protein from each sample were assessed using SDS–PAGE with Tris-Glycine gels and then transferred to polyvinylidene fluoride membranes. Then, 5% bovine serum albumin in Tris-buffered saline containing 0.1% Tween 20 (TBST) was used to block the membranes, which were then incubated overnight with primary antibodies at 4°C. The following primary antibodies were used: cleaved-Caspase-3 (Cell Signaling Technology, 9661), Bcl-2 (Abcam, ab59348), Bax (Abcam, ab32503), c-Myc (Abcam, ab32072), SIRT1 (Cell Signaling Technology, 8469), forkhead box O1 (FOXO1; Cell Signaling Technology, 2880), and PCNA (Abcam, ab29). After incubation, each membrane was washed with TBST three times for 5 min and then incubated at room temperature in either HRP-goat-anti-rabbit (Abcam, ab6721) or HRP-goat-anti-mouse (Abcam, ab6789) secondary antibodies for 1 h. The Pierce ECL plus western blotting substrate (Thermo Scientific, 32132) was used to visualize the resulting immunoreactive bands. Antigen expression levels were individually quantified using ImageQuant 5.2 software (Molecular Dynamics), with GAPDH (Abcam, ab9485) and Lamin B1 (Cell Signaling Technology, 12586) expression levels used as loading controls.

### RNA Isolation and Semi-Quantitative RT-PCR (sqRT-PCR)

TRIzol Reagent (Invitrogen, 15596018) was used to extract total RNA from HUVECs. Then, 2 μg of the resulting total RNA was reverse transcribed into cDNA using the GoScript Reverse Transcription Kit (Promega, A5001). The cDNAs were then subjected to sqRT-PCR analysis, in which gene expression was quantified using previously described methods (Magalhães et al., 2013). The mRNA levels of target genes were normalized using *GAPDH* expression. The following gene-specific primer sequences were used for qRT-PCR:

FOXO1

Sense 5′- AACCT GGCATTACAGTTGGCC -3′

Antisense 5′- AAATGCAGGAGGCATGACTACGT -3′

SIRT1

Sense 5′- GCCTCACATGCAAGCTCTAGTGAC -3′

Antisense 5′- TTCGAGGATCTGTGCCAATCATAA -3′

GAPDH

Sense 5′- GACCTGCCGTCTAGAAAAAC -3′

Antisense 5′- CTGTAGCCAAATTCGTTGTC -3′

### Terminal Deoxynucleotidyl Transferase-Mediated dUTP Nick End Labeling Assay

Terminal deoxynucleotidyl transferase-mediated dUTP nick end labeling (TUNEL) assay was performed by fixing HUVECs in PBS containing 4% PFA, and an aortic ring from each mouse was fixed in PBS containing 4% PFA and then embedded in paraffin. Slides of the paraffinized tissues were prepared after being sectioned into 5-μm-thick slices. The *In situ* Cell Death Detection kit (Roche, 11684795910) was used for staining according to the manufacturer’s protocol.

### Dihydroethidium Staining and ATP Synthesis Assay

Intracellular ROS production was measured by dihydroethidium (DHE) staining. The staining was processed for imaging under a fluorescence microscope. Cellular ATP levels were measured with a kit of ATP assay (Beyotime, S0026) as described previously (Sun et al., 2019).

### Mitochondrial Respiration Assay

The oxygen consumption rate (OCR) of HUVECs was measured in intact cells with the XF24 analyzer from Seahorse Bioscience (Billerica, MA, United States) as described previously (Kandasamy et al., 2017).

### Immunofluorescence Staining of HUVECs and Aortic Ring Sections

Human umbilical vein endothelial cells were grown on gelatinized coverslips overnight. Briefly, after the experimental period described above, HUVECs were fixed in 4% paraformaldehyde in PBS for 10 min, and permeabilized with 0.5% Triton for 15 min. The cells were then incubated overnight with PCNA antibody (Abcam, ab29) and/or Ki67 (Cell Signaling Technology, 11882), followed by a 1 h incubation with or without the Alexa Fluor 488-conjugated anti-mouse IgG secondary antibody (Abcam, ab150113) at room temperature. With another 1 h incubation, HUVEC nuclei were labeled with the fluorescent dye DAPI. All observations of cells under each experimental condition were conducted using a TCS SP5 Confocal microscope (Leica, Wetzlar, Germany).

For aortic ring staining, 5-μm-thick paraffin sections were cut and incubated with anti-CD31 (Abcam, ab24590) and/or PCNA (Abcam, ab29). Samples were washed and then incubated at room temperature for 1 h with Alexa Fluor 647-conjugated anti-rabbit IgG secondary antibody and/or Alexa Fluor 488-conjugated anti-mouse IgG secondary antibody at a 1:200 dilution. DAPI was used to label cell nuclei. Immunofluorescent staining were also subjected to incubation with either mouse IgG isotype control (Cell Signaling Technology, 5415) or rabbit IgG isotype control (Cell Signaling Technology, 3900) according to the immunoglobulin type of primary antibody, thus serving as the key primary antibody control (Supplementary Figure S1A). A TCS SP5 Confocal microscope (Leica) was used to digitally capture images.

### Wound Healing Assay

A previously described wound healing scratch assay was used to assess cell migration (Das et al., 2018). Cells were plated onto a 3.5-cm-diameter dish and cultured overnight until a confluent monolayer was formed, into which a scratch was made with a 200-μL pipette tip. The effects of HG, 10 μM RES, si-*SIRT1*, and Ad-*FOXO1* on wound healing were measured 12, 24, and 36 h after wounding. An IX70 microscope (Olympus, Tokyo, Japan) equipped with a CoolSNAP HQ CCD camera (Nippon Roper, Chiba, Japan) and controlled by MetaMorph software (Universal Imaging Co., Ltd., United Kingdom) was used to capture images of the wounded cell monolayers at 0, 12, 24, and 36 h after wounding and also to record picture for 36 h. Cell proliferation was inhibited in all experiments using 5 mg/mL of mitomycin-C.

### Transfection of siRNA Into Cells

RNA interference was carried out by transfecting cells with *c-Myc* siRNA (Santa Cruz Biotechnology, sc-29226), *SIRT1* siRNA (Santa Cruz Biotechnology, sc-40986), or control scrambled siRNA (Santa Cruz Biotechnology, sc-37007) using Lipofectamine 2000 for 12 h in Opti-MEM according to the manufacturer’s instructions. After transfection, cells were transferred to full-growth medium for another 12-h incubation prior to analysis for further studies. HUVECs were treated with HG (33 mM) for 72 h with or without 10 μM RES.

### *In vivo* Wound Model

General anesthesia was performed with 2% inhaled isoflurane and then injected subcutaneously with the analgesic. After the hair on the backs of mice was removed using an electric clipper, depilatory cream was applied. Alcohol was used to rinse the skin, and two full-thickness wounds were created using a 3-mm biopsy punch on the dorsum on each side of the midline. One wound was smeared with 10 μM RES (Spallotta et al., 2013; Zhao et al., 2017), while the second wound (internal control) received 50 μL of sterile PBS. For signaling pathway analysis, the wound smeared with RES received either Ad-*FOXO1* solution (1 × 10^8^ PFU prepared in 50 μL of PBS) or EX-527 (10 μM) or 10058-F4 (50 μM). The wounds were harvested 7 d post-wounding. Wounds were bisected on the longitudinal diameter of the wound and fixed in 10% neutral buffered formalin for CD31 immunofluorescence analyses. Ad-*FOXO1* solution was injected intradermally into the wound edges of the mice the day before wounding (Li et al., 2015), and both immediately and 4 days after wounding, inhibitors were intradermally injected into the wound edges. Images of the wound areas were captured, and the wounds were measured every other day until approximately 90% of the wound areas had healed.

### Immunofluorescence Staining of Mouse Wound Tissue

Incubations with anti-CD31 (Abcam, ab24590), Alexa Fluor 488-conjugated KI67 (Cell Signaling Technology, 11882), or Alexa Fluor 488-cleaved-Caspase-3 (Cell Signaling Technology, 9669) were conducted with 5-μm-thick paraffin sections. Samples were then washed and incubated at room temperature for 60 min with a 1:200 dilution of Alexa Fluor 647-conjugated anti-rabbit IgG secondary antibody DAPI was used to label cell nuclei. Immunofluorescent staining were also subjected to incubated with rabbit IgG isotype control (Cell Signaling Technology, 3900) according to the immunoglobulin type of primary antibody, thus serving as the key primary antibody control (Supplementary Figure S1A). A TCS SP5 Confocal microscope (Leica) was used to capture digital images.

### Blood Glucose and Plasma Insulin Levels Assay

After withholding food for12 h blood samples were obtained from the tail veins, and blood glucose level was measured by glucose analyzer (Lifescan Surestep). Plasma insulin levels were assessed using an UltraSensitive Mouse Insulin ELISA kit (ALPCO Diagnostics).

### Adenovirus Constructs

A previously described protocol was used for the construction, propagation, and titration of recombinant adenovirus vectors as previously described (Maejima et al., 2013). In short, pBHGloxΔE1,3 Cre plasmid (Microbix, PD-01-40), which includes the entire ΔE1 adenoviral genome, was co-transfected along with the pDC316 shuttle vector, which contained *FOXO1*, the gene of interest into HEK293 cells using Lipofectamine 2000 (Life Technologies, 11668019). The test genes were integrated into the E1-deleted adenoviral genome via homologous recombination. The transformed viruses were then propagated into HEK293 cells. This produced replication-defective human adenovirus type 5 (devoid of E1) harboring human *FOXO1*.

### Statistical Analyses

Results were expressed as mean ± SEM values, and statistically significant differences were determined using unpaired 2-tailed Student’s *t*-tests for comparisons between two experimental groups and one-way ANOVA for comparisons among multiple groups, using SPSS. Bonferroni’s *post hoc* tests were employed to assess significant differences among groups. A two-tailed *p*-value of less than 0.05 was considered statistically significant. Statistical analyses were conducted with GraphPad Prism (GraphPad Software).

## Results

### RES Attenuates Hyperglycemia-Induced Endothelial Dysfunction *in vivo* and *in vitro*

The db/db mice we used exhibited dramatic increases in both fasting blood glucose and plasma insulin levels. Systemic treatment of diabetic db/db mice using 50 mg/kg/day doses of RES could lower the fasting blood glucose and plasma insulin levels (Supplementary Figures S1B,C). The protective effects of RES against hyperglycemia-induced endothelial impairment were demonstrated *in vivo* using immunofluorescence staining for CD31. This revealed deendothelialized regions in the aortic endothelium of db/db mice that were absent in their corresponding control db/m littermates. RES significantly attenuated the hyperglycemia-induced de-endothelialization relative to the vehicle-treated group (Supplementary Figures S2A,B). The proliferation of aortic endothelial cells was examined by immunofluorescence staining with proliferating cell nuclear antigen (PCNA). RES treatment increased hyperglycemia-impaired endothelial cell proliferation, which showed an increased number of PCNA-positive endothelial cells compared with db/db mice (Supplementary Figures S2C,D). Moreover, the aortic endothelium of db/db mice exhibited a high level of apoptosis, as reflected by TUNEL staining, while RES ameliorated hyperglycemia-induced endothelial apoptosis (Supplementary Figures S2E,F).

**FIGURE 1:**
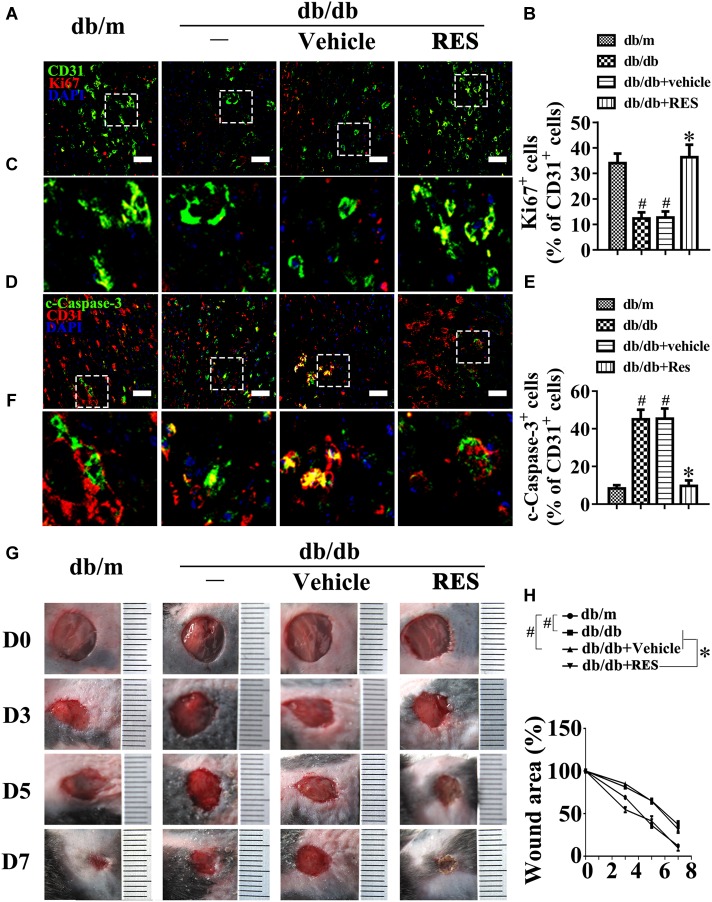
Resveratrol (RES) attenuates hyperglycemia-induced endothelial dysfunction *in vivo*. **(A)** The presence of immunofluorescence with CD31 and Ki67 of wounded skin tissue sections, scale bars = 30 μm (×400), **(C)** enlargement of the indicated area in **(A)**, scale bars = 12 μm (×1000), **(D)** confocal immunofluorescence with CD31 and c-Caspase-3 of wounded skin tissue sections, scale bars = 30 μm (×400), **(F)** enlargement of the indicated area in **(D)**, scale bars = 12 μm (×1000), and **(G)** images of skin wounds, from db/m mice, db/db mice and db/db mice receiving RES (10 μM) or vehicle treatment with saline smeared on the wound, *n* = 6 mice in each group. Quantification of the proportion of Ki67 positive staining of endothelial cells **(B)**, c-Caspase-3 positive staining of endothelial cells **(E)** and wound areas **(H)**. All values displayed are means ± SEM of 8 independent experiments. ^#^*p* < 0.05 vs. db/m mice; ^∗^*p* < 0.05 vs. db/db mice or vehicle treated db/db mice.

We examined the functional role of RES *in vivo* with a skin wound healing model in mice with type 2 diabetes mellitus (T2DM), the pathogenesis of which involves endothelial cells. Topical administration of RES in diabetic mice skin wounds hardly had an influence on the fasting blood glucose and plasma insulin levels (Supplementary Figures S1D,E). But we observed that CD31^+^ capillary density decreased in the regenerative skin tissue in the wounds of db/db mice, which was restored by RES treatment (Supplementary Figures S2G,H). Furthermore, the Ki67 positive endothelial cells largely increased, which correlated with higher blood vessel density due to RES treatment, suggesting that RES could promote endothelial cells proliferation during diabetic skin wound healing (Figure 1A–C). Meanwhile, the apoptosis of endothelial cells were also alleviated in RES treated regenerative skin in the wounds of db/db mice as revealed by a dramatically decrease in the c-Caspase-3 immunofluoresent intensity (Figure 1D–F). Accordingly, RES treatment largely accelerated wound healing in db/db mice (Figure 1G,H).

**FIGURE 2:**
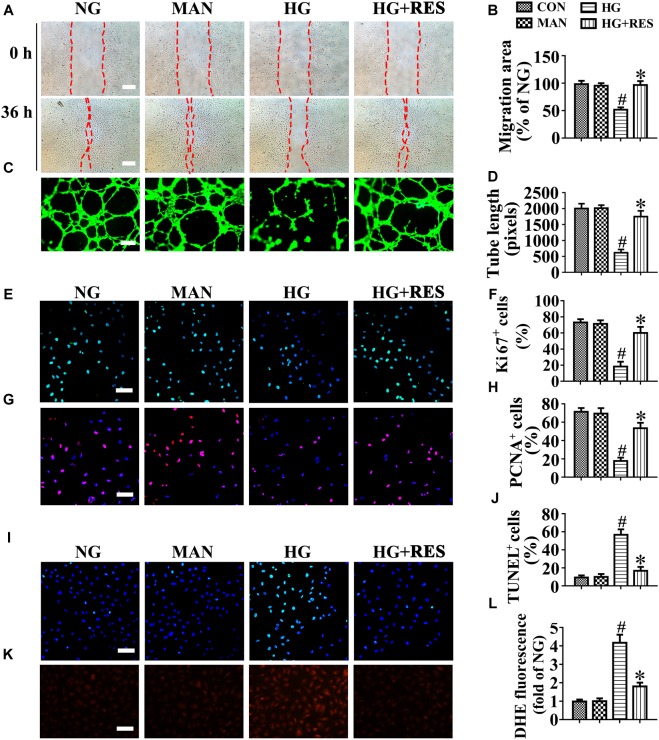
Resveratrol attenuates hyperglycemia-induced endothelial dysfunction *in vitro*. **(A)** The presence of human umbilical vein endothelial cells (HUVECs) wound healing assay, scale bars = 300 μm (×25), **(C)** capillary-like tube formation, scale bars = 300 μm (×25), **(E)** immunofluorescence with Ki67, scale bars = 100 μm (×200), **(G)** immunofluorescence with PCNA, scale bars = 100 μm (×200), **(I)** TUNEL assay, scale bars = 100 μm (×200), and **(K)** fluorescence with DHE, scale bars = 100 μm (×200), HUVECs were cultured in different mediums containing NG (5.5 mM), HG (33 mM) alone, or with RES (10 μM) for 72 h, mannitol (MAN; 33 mM: 5.5 mM of glucose + 27.5 mM of _D_-mannitol) was served as the osmotic control for the HG. Quantification of the cell migration distance **(B)**, the tube length **(D)**, the Ki67 fluorescence intensity ratio **(F)**, the PCNA fluorescence intensity ratio **(H)**, the quantitative analysis of TUNEL^+^ cells **(J)**, the DHE fluorescence intensity ratio **(L)**. All values displayed are means ± SEM of 8 independent experiments. ^#^*p* < 0.05 vs. NG or MAN; ^∗^*p* < 0.05 vs. HG.

To further assess endothelial function, an *ex vivo* model, that is, an aortic ring assay, was employed. C57BL/6 mouse aortic rings were cultured in media containing NG (5.5 mM), HG (33 mM), or HG (33 mM) with RES. The aortic rings cultured in NG medium exhibited a well-structured microvessel network characterized by regular branching. However, those cultured in HG medium were observed to exhibit dramatically impaired sprouting relative to rings in the osmotic control group or cultured in normal medium, though sprouting was restored under RES treatment (Supplementary Figures S2I,J). Meanwhile, migration (Figure 2A,B) and tube-forming activity (Figure 2C,D) were also significantly impaired in HUVECs in the HG treatment. Furthermore, immunofluorescence staining with Ki67 (Figure 2E,F) and PCNA (Figure 2G,H) as well as immunoblotting for PCNA proteins (Supplementary Figures S2K,N) demonstrated the impaired proliferation of HUVECs in HG, which was greatly improved by RES co-treatment. In addition, the HG treatment induced high levels of apoptosis in HUVECs, as demonstrated by increased numbers of TUNEL-positive cells (Figure 2I,J) and elevated levels of cleaved-Caspase-3 protein (Supplementary Figures S2K,M), as well as an increased Bax/Bcl-2 ratio (Supplementary Figures S2K,L). However, HG-induced apoptosis was significantly alleviated by RES. Besides, HG exposure largely increased the ROS level in cultured HUVECs, and induced a significance decrease in ATP production compared to the NG group. However, RES treatment significantly inhibited HG-induced ROS generation (Figure 2K,L) and change in ATP production (Supplementary Figures S4H,J).

Furthermore, we assessed whether RES could play a role in mitochondrial dysfunction. Thus we assessed the mitochondrial OCR. A mitochondrial respiratory reserve capacity (maximal OCR over baseline OCR) was significantly reduced in HG-treated HUVECs as compared to NG. In contrast, a reverse respiratory capacity was found in HG-treated cells with RES addition (Supplementary Figures S4G,I). Taken together, these data confirmed the protective role of RES against HG-induced endothelial impairment.

### The Endothelial Protective Action of RES Against Hyperglycemia Is SIRT1-Dependent

**FIGURE 3:**
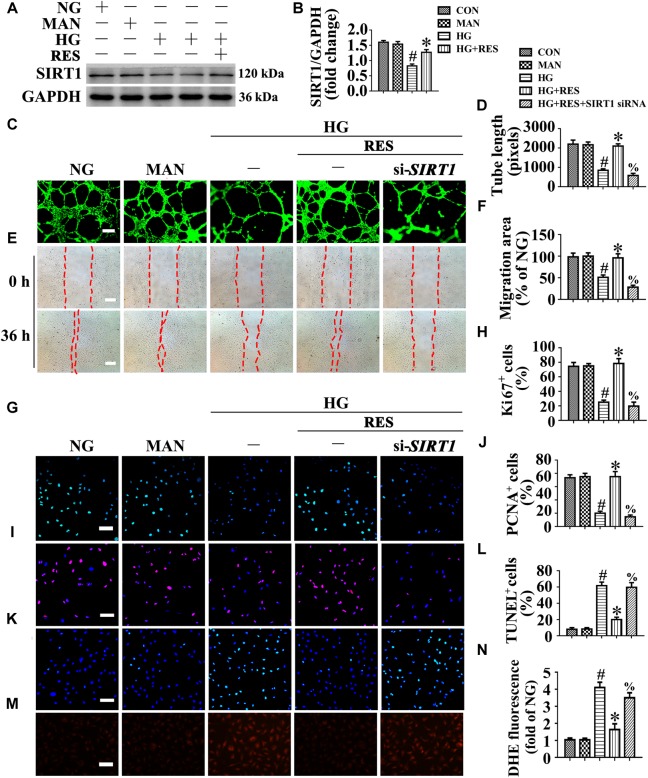
The endothelial protective action of RES against HG is silent information regulator 1 (SIRT1) dependent, *in vitro*. Cell lysates of HUVECs were used to detect **(A)** the SIRT1 protein levels by immunoblotting. HUVECs were cultured either in NG or HG medium in the presence or absence of RES (10 μM) for 72 h, MAN was served as the osmotic control for the HG. **(B)** The quantitative analysis of immunoblot. **(C)** HUVECs capillary-like tube formation, scale bars = 300 μm (×25), **(E)** wound healing assay, scale bars = 300 μm (×25), **(G)** immunofluorescence with Ki67, scale bars = 100 μm (×200), **(I)** immunofluorescence with PCNA, scale bars = 100 μm (×200), **(K)** TUNEL assay, scale bars = 100 μm (×200), and **(M)** fluorescence with DHE, scale bars = 100 μm (×200), HUVECs were transduced with transfected with *SIRT1* siRNA or control siRNA, respectively. After transduction, HUVECs were cultured either in NG or HG medium alone or with RES (10 μM) for 72 h. Quantification of the tube length **(D)**, the cell migration distance **(F)**, the Ki67 fluorescence intensity ratio **(H)**, the PCNA fluorescence intensity ratio **(J)**, the quantitative analysis of TUNEL^+^ cells **(L)**, the DHE fluorescence intensity ratio **(N)**. All values displayed are means ± SEM of 8 independent experiments. ^#^*p* < 0.05 vs. corresponding NG; ^∗^*p* < 0.05 vs. HG; ^%^*p* < 0.05 vs. HG co-incubated with RES.

A growing body of evidence supports an important role for SIRT1 in vascular homeostasis and repair in diabetic individuals (Zhou et al., 2011; Hammer et al., 2017). Consistent with previous studies (Zhou et al., 2011; Yerra et al., 2017), our results confirmed the presence of impaired SIRT1 protein expression in HUVECs exposed to HG. Importantly, RES largely reversed HG-downregulated SIRT1 expression (Figure 3A,B).

We explored whether the endothelial protective effect of RES was SIRT1-dependent. *SIRT1* was deleted in HUVECs by siRNA transfection. The results demonstrated that the endothelial protective effect of RES against HG was abolished in *SIRT1*-deficient HUVECs, which exhibited disrupted tube forming activity (Figure 3C,D) and increased levels of apoptosis (Figure 3K,L). In si-*SIRT1*-transfected HUVECs, the RES-mediated pro-migration (Figure 3E,F) and pro-proliferation (Figure 3G–J) effects against HG impairment were abrogated. Meanwhile, *SIRT1* knockdown also elevated the ROS level (Figure 3M,N) and induced a significance decrease in ATP production in RES with HG cultured HUVECs (Supplementary Figure S4J). Moreover, OCR assessment also demonstrated a reduced mitochondrial respiratory reserve capacity due to *SIRT1* knockdown in RES with HG treated HUVECs (Supplementary Figure S4I). Pretreatment with EX-527, a specific SIRT1 inhibitor, also abrogated the RES-mediated anti-apoptosis (Supplementary Figures S3I–K) and pro-proliferation (Supplementary Figures S3I,L) effects against HG in HUVECs.

Moreover, we generated endothelium-specific *SIRT1* knockout mice by mating *SIRT1*-floxed mice (*SIRT1* flox/flox) with mice expressing *Cre* recombinase under the control of the endothelial-specific promoter *Tie2*. *SIRT1* knockdown was confirmed by immunoblotting and sqRT-PCR for SIRT1 in aortic homogenates from *SIRT1* flox/flox; *Tie2-Cre* (+) mice and their control littermates, *SIRT1* flox/flox; *Tie2-Cre* (-) mice (Supplementary Figures S3M–O). Then, we performed the aortic ring assay. Similar with the results obtained in the *in vitro* studies, RES attenuated the HG-impaired sprouting of aortic rings from *SIRT1* flox/flox; *Tie2-Cre* (-) mice; However, this endothelial protective effect was abolished in *SIRT1* flox/flox; *Tie2-Cre* (+) mice (Supplementary Figures S3P,Q).

In the aortic endothelium of db/db mice, we observed that the RES-induced pro-endothelialization (Supplementary Figures S3A,B) and pro-proliferation (Supplementary Figures S3C,D) effects were diminished by systemic EX-527 treatment, along with increased apoptosis in the endothelium (Supplementary Figures S3E,F). But systemic EX-527 treatment could not reverse RES downregulated fasting blood glucose and plasma insulin levels (Supplementary Figures S1B,C).

**FIGURE 4:**
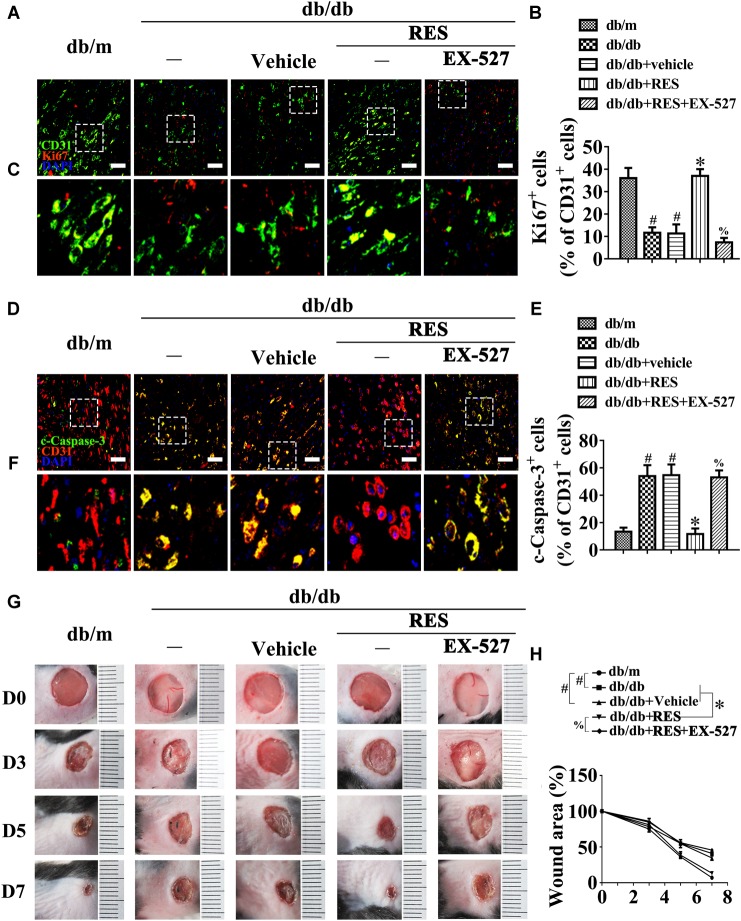
The endothelial protective action of RES against HG is SIRT1 dependent, *in vivo*. **(A)** The presence of immunofluorescence with CD31 and Ki67 of wounded skin tissue sections, scale bars = 30 μm (×400), **(C)** enlargement of the indicated area in **(A)**, scale bars = 12 μm (×1000), **(D)** confocal immunofluorescence with CD31 and c-Caspase-3 of wounded skin tissue sections, scale bars = 30 μm (×400), **(F)** enlargement of the indicated area in **(D)**, scale bars = 12 μm (×1000), and **(G)** images of skin wounds, from db/m mice, db/db mice and db/db mice receiving RES (10 μM) or vehicle treatment with saline smeared on the wound, *n* = 6 mice in each group. For signaling pathway analysis, EX-527 (10 μM) was injected intradermally into the wound edges in the mice after RES smeared on the wound. Quantification of the proportion of Ki67 positive staining of endothelial cells **(B)**, c-Caspase-3 positive staining of endothelial cells **(E)**, and wound areas **(H)**. All values displayed are means ± SEM of 8 independent experiments. ^#^*p* < 0.05 vs. db/m mice; ^∗^*p* < 0.05 vs. db/db mice or vehicle treated db/db mice; % *p* < 0.05 vs. db/db mice receiving RES.

We then examined the functional role of RES-mediated SIRT1 activation *in vivo* using the skin wound healing model with T2DM mice. Topical administration of EX-527 in diabetic mice skin wounds hardly had an influence on the fasting blood glucose and plasma insulin levels (Supplementary Figures S1D,E). We observed that topic administration of RES-restored proliferation of endothelial cells along with CD31^+^ capillary density in diabetic regenerative skin tissue, but was abrogated by topical EX-527 treatment (Figure 4A–C). Meanwhile, RES-inhibited endothelial cell apoptosis was also abolished due to EX-527 treatment (Figure 4D–F) (Supplementary Figures S3G,H). As a result, RES-accelerated wound healing in db/db mice was abrogated by EX-527 (Figure 4G,H). These results demonstrated that the endothelial protective action of RES against hyperglycemia is SIRT1-dependent.

### FOXO1, as a Downstream Molecule of SIRT1, Participates in the Endothelial Protective Action of RES Against Hyperglycemia

**FIGURE 5:**
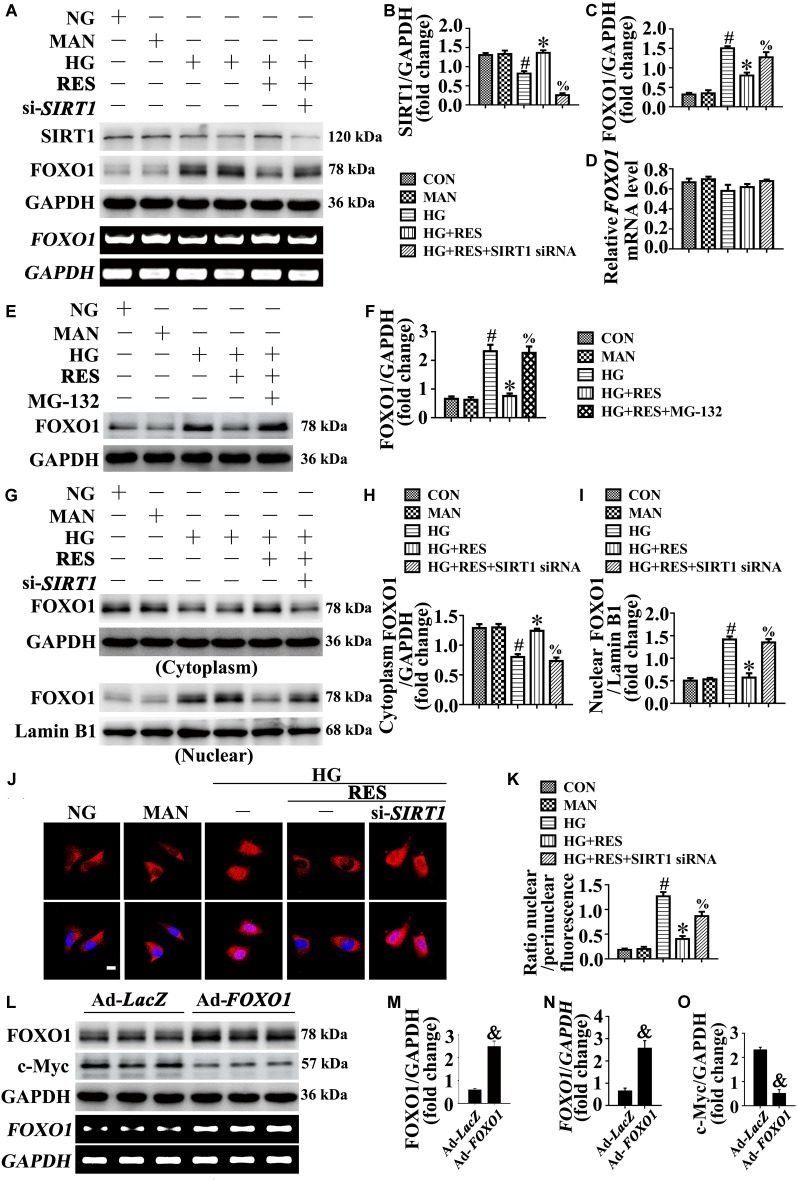
FOXO1 participates in the endothelial protective action of RES against hyperglycemia, *in vitro*. **(A)** Cell lysates of HUVECs were used to detect the SIRT1 and FOXO1 protein levels by immunoblotting. sqRT-PCR analysis of *FOXO1* mRNA level in HUVECs. HUVECs were transduced with *SIRT1* siRNA or control siRNA, respectively. After transduction, HUVECs were cultured either in NG or HG medium alone or with RES (10 μM) for 72 h, MAN was served as the osmotic control for the HG. **(B**–**D)** The quantitative analysis of **(A)**. **(E)** Cell lysates of HUVECs were used to detect the FOXO1 protein levels by immunoblotting. HUVECs were cultured either in NG or HG medium alone or with RES (10 μM) for 72 h, MAN was served as the osmotic control for the HG. For signaling pathway analysis, MG-132 (0.5 μM) was pretreated for 2 h before RES administration. **(F)** The quantitative analysis of **(E)**. **(G)** Nuclear and cytosolic extracts were isolated to detect the FOXO1 protein levels by immunoblotting. HUVECs treated as indicated in **(A)**. **(H,I)** The quantitative analysis of each immunoblots. **(J)** Representative immunofluorescence with FOXO1 in HUVECs, which treated as indicated in **(A)**. Scale bars = 5 μm (×400). **(K)** The quantitative analysis of nuclear/perinuclear FOXO1 fluorescence intensity ratio in **(J)**. **(L)** Cell lysates of HUVECs were used to detect the FOXO1 and c-Myc protein levels by immunoblotting. sqRT-PCR analysis of FOXO1 mRNA level in HUVECs. HUVECs were transduced with Ad-*FOXO1* and Ad-*LacZ*, respectively. After transduction, HUVECs were cultured in NG. **(M**–**O)** The quantitative analysis of **(L)**. All values displayed are means ± SEM of 8 independent experiments. ^#^*p* < 0.05 vs. NG or MAN; ^∗^*p* < 0.05 vs. HG; ^%^*p* < 0.05 vs. HG co-incubated with RES; ^&^*p* < 0.05 vs. Ad-*LacZ* transducing HUVECs cultured in NG.

Forkhead box O1 transcription factors mediate the pro-angiogenic function of SIRT1 (Potente et al., 2007). FOXO1 is an important substrate of SIRT1, which is a critical checkpoint of endothelial growth that restricts vascular expansion (Wilhelm et al., 2016). In HG-exposed HUVECs, we observed decreased SIRT1 protein expression (Figure 5A,B), along with increased FOXO1 expression (Figure 5A,C), compared with NG. RES with HG co-treatment greatly increased SIRT1 protein expression and downregulated the level of FOXO1 protein. To demonstrate that the RES-modulated downregulation of FOXO1 was attributed to the increased expression of SIRT1 against HG, HUVECs were transfected with siRNA targeting *SIRT1*. In *SIRT1*-deficient HUVECs, RES could no longer downregulate FOXO1 expression against HG. Meanwhile the transcription level of FOXO1 was unchanged in HUVECs treated with HG and in HUVECs co-incubated with HG and RES (Figure 5A,D). The proteasome inhibitor MG-132 restored the RES-decreased FOXO1 protein level (Figure 5E,F), thus confirming our speculation that RES-modulated FOXO1 downregulation was attributed to increased protein degradation against HG.

We further analyzed the subcellular localization of FOXO1. The nucleus and cytoplasm of HUVECs were separated and analyzed using an immunoblotting assay. In HG-incubated HUVECs, we found that FOXO1 was mainly localized in the nucleus, while it was distributed in the cytoplasm after co-incubation with RES (Figure 5G–I). However, in si-*SIRT1*-transfected HUVECs, the RES-mediated cytoplasmic distribution of FOXO1 was abolished under HG. The subcellular localization of FOXO1 was also confirmed by immunofluorescence staining (Figure 5J,K).

**FIGURE 6:**
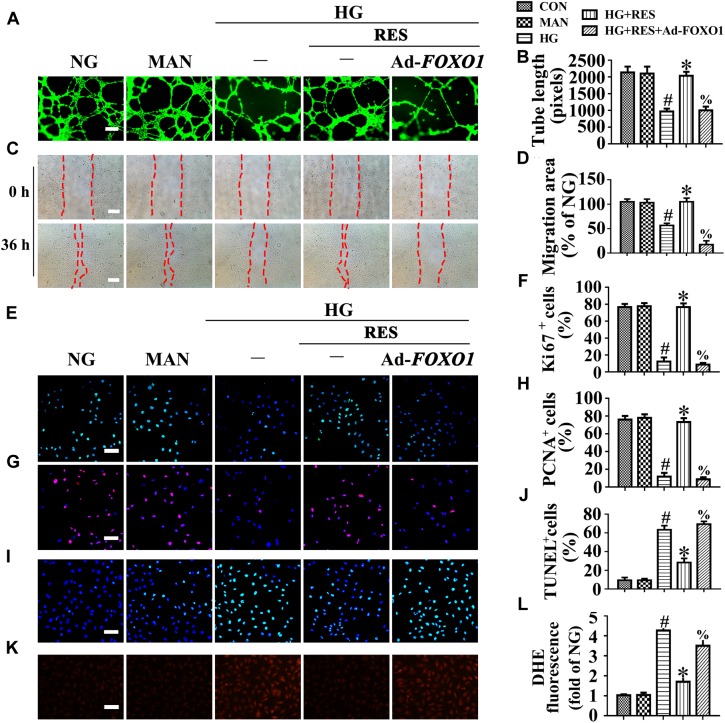
FOXO1 participates in the endothelial protective action of RES against hyperglycemia, *in vitro*. **(A)** HUVECs capillary-like tube formation, scale bars = 300 μm (×25), **(C)** wound healing assay, scale bars = 300 μm (×25), **(E)** immunofluorescence with Ki67, scale bars = 100 μm (×200), **(G)** immunofluorescence with PCNA, scale bars = 100 μm (×200), **(I)** TUNEL assay, scale bars = 100 μm (×200), **(K)** fluorescence with DHE, scale bars = 100 μm (×200), HUVECs were transduced with Ad-*FOXO1* and Ad-*LacZ*, respectively. After transduction, HUVECs were cultured either in NG or HG medium in the presence or absence of RES (10 μM) for 72 h, MAN was served as the osmotic control for the HG. Quantification of the tube length **(B)**, the cell migration distance **(D)**, the Ki67 fluorescence intensity ratio **(F)**, the PCNA fluorescence intensity ratio **(H)**, the quantitative analysis of TUNEL^+^ cells **(J)**, the DHE fluorescence intensity ratio **(L)**. All values displayed are means ± SEM of 8 independent experiments. ^#^*p* < 0.05 vs. NG or MAN; ^∗^*p* < 0.05 vs. HG; ^%^*p* < 0.05 vs. HG co-incubated with RES.

To further demonstrate the critical role of FOXO1 in RES-mediated endothelial protection under high glucose conditions, FOXO1 was overexpressed in HUVECs by adenovirus. Forced FOXO1 overexpression had a significant influence on the c-Myc protein stability, resulting in the degradation of c-Myc protein (Figure 5L–O). FOXO1 overexpression abolished RES-mediated endothelial protection against HG, while it also disrupted tube formation (Figure 6A,B) and increased the level of apoptosis (Figure 6I,J and Supplementary Figures S4C–E). In FOXO1-overexpressing HUVECs, the RES-induced pro-migration (Figure 6C,D) and pro-proliferation (Figure 6E–H and Supplementary Figures S4C,F) effects against HG impairment were also abrogated. Meanwhile, FOXO1 overexpression also elevated the ROS level (Figure 6K,L) and induced a significance decrease in ATP production in RES with HG cultured HUVECs (Supplementary Figure S4H). Moreover, OCR assessment also demonstrated a reduced mitochondrial respiratory reserve capacity due to FOXO1 overexpression in RES with HG treated HUVECs (Supplementary Figure S4G).

**FIGURE 7:**
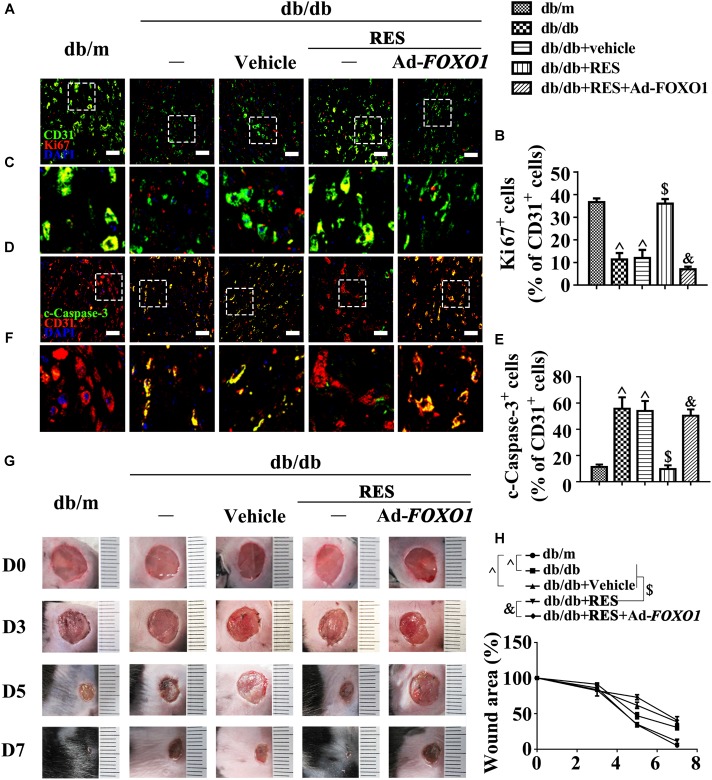
FOXO1 participates in the endothelial protective action of RES against hyperglycemia. **(A)** The presence of immunofluorescence with CD31 and Ki67 of wounded skin tissue sections, scale bars = 30 μm (×400), **(C)** enlargement of the indicated area in **(A)**, scale bars = 12 μm (×1000), **(D)** confocal immunofluorescence with CD31 and c-Caspase-3 of wounded skin tissue sections, scale bars = 30 μm (×400), **(F)** enlargement of the indicated area in **(D)**, scale bars = 12 μm (×1000), and **(G)** images of skin wounds, from db/m mice, db/db mice and db/db mice receiving RES (10 μM) or vehicle treatment with saline smeared on the wound, *n* = 6 mice in each group. For signaling pathway analysis, Ad-*FOXO1* was injected intradermally into the wound edges in the mice the day before wounding. Quantification of the proportion of Ki67 positive staining of endothelial cells **(B)**, c-Caspase-3 positive staining of endothelial cells **(E)** and wound areas **(H)**. All values displayed are means ± SEM of 8 independent experiments. ^∧^*p* < 0.05 vs. db/m mice; ^$^*p* < 0.05 vs. db/db mice or vehicle treated db/db mice; ^&^*p* < 0.05 vs. db/db mice receiving RES.

These results were confirmed *in vivo* using the skin wound healing model with T2DM mice. Local delivery of adenovirus-mediated FOXO1 overexpression in diabetic mice skin wounds hardly had an influence on the fasting blood glucose and plasma insulin levels (Supplementary Figures S1D,E). But we observed that RES-restored proliferation of endothelial cells along with CD31^+^ capillary density was abrogated by FOXO1 overexpression in diabetic regenerative skin tissue (Figure 7A–C). Meanwhile, RES-inhibited endothelial cell apoptosis was also abolished due to FOXO1 overexpression (Figure 7D–F) (Supplementary Figures S4A,B). As a result, RES-accelerated wound healing in db/db mice was abrogated by FOXO1 overexpression (Figure 7G,H).

### c-Myc, as the Main Effector of FOXO1, Participates in the Endothelial Protective Action of RES Against Hyperglycemia

**FIGURE 8:**
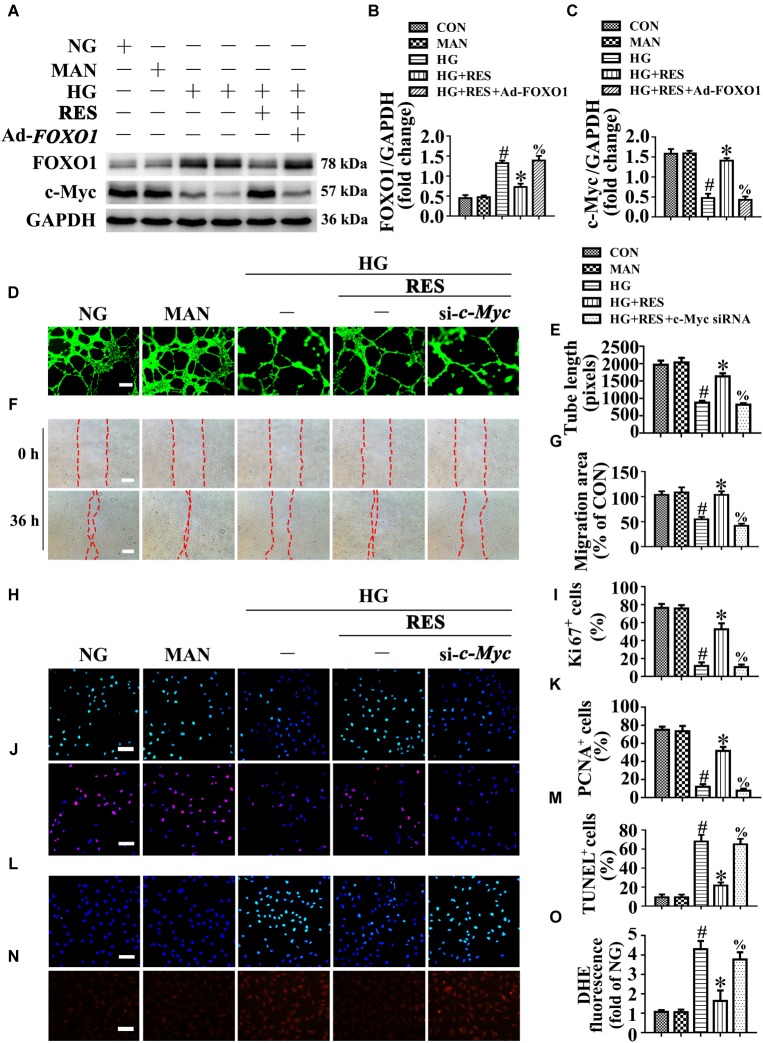
c-Myc participates in the endothelial protective action of RES against hyperglycemia *in vitro*. **(A)** Cell lysates of HUVECs were used to detect the FOXO1 and c-Myc protein levels by immunoblotting. HUVECs were transduced with Ad-*FOXO1* and Ad-*LacZ*, respectively. After transduction, HUVECs were cultured either in NG or HG medium in the presence or absence of RES (10 μM) for 72 h. **(B**,**C)** The quantitative analysis of each immunoblots. **(D)** HUVECs capillary-like tube formation, scale bars = 300 μm (×25), **(F)** wound healing assay, scale bars = 300 μm (×25), **(H)** immunofluorescence with Ki67, scale bars = 100 μm (×200), **(J)** immunofluorescence with PCNA, scale bars = 100 μm (×200), **(L)** TUNEL assay, scale bars = 100 μm (×200), **(N)** fluorescence with DHE, scale bars = 100 μm (×200), HUVECs were transduced with transfected with *c-Myc* siRNA or control siRNA, respectively. After transduction, HUVECs were cultured either in NG, or HG medium alone or with RES (10 μM) for 72 h. Quantification of the tube length **(E)**, the cell migration distance **(G)**, the Ki67 fluorescence intensity ratio **(I)**, the PCNA fluorescence intensity ratio **(K)**, the quantitative analysis of TUNEL^+^ cells **(M)**, the DHE fluorescence intensity ratio **(O)**. All values displayed are means ± SEM of 8 independent experiments. ^#^*p* < 0.05 vs. corresponding NG; ^∗^*p* < 0.05 vs. HG; ^%^*p* < 0.05 vs. HG co-incubated with RES.

We sought to gain insight into the underlying mechanism of the FOXO1-mediated depression of endothelial function under high glucose conditions. A previous study reported that FOXO1 antagonizes endothelial c-Myc signaling (Wilhelm et al., 2016), and because c-Myc is a powerful driver of glycolysis, mitochondrial metabolism, and growth, we hypothesized that RES induce FOXO1 inactivation by activating SIRT1, to restore hyperglycemia-triggered endothelial dysfunction and disturbance of angiogenesis, followed by the promotion of diabetic wound healing in a c-Myc-dependent manner. In line with this, we observed that c-Myc expression was decreased in HUVECs under HG, where FOXO1 was highly expressed. RES inhibited HG-induced FOXO1 expression but upregulated c-Myc expression. Furthermore, FOXO1 overexpression in RES with HG-treated HUVECs suppressed c-Myc expression (Figure 8A–C).

To confirm the functional role of c-Myc in RES-mediated endothelial protection, *c-Myc* was deleted in HUVECs by siRNA transfection. The results showed that the endothelial protective effects of RES against HG were abolished in *c-Myc*-deficient HUVECs, tube formation was disrupted (Figure 8D,E), and apoptosis was increased (Figure 8L,M). In si-*c-Myc*-transfected HUVECs, the RES-induced pro-migration (Figure 8F,G) and pro-proliferation (Figure 8H–K) effects against HG impairment were also abrogated. Meanwhile, deletion of *c-Myc* elevated the ROS level (Figure 8N,O) and induced a significance decrease in ATP production in RES with HG cultured HUVECs (Supplementary Figure S4J). Moreover, OCR assessment demonstrated a reduced mitochondrial respiratory reserve capacity due to *c-Myc* deletion in RES with HG treated HUVECs (Supplementary Figure S4I).

Pretreatment with 10058-F4, a specific c-Myc inhibitor, abrogated the RES-mediated anti-apoptosis (Supplementary Figures S5I–K) and pro-proliferation (Supplementary Figures S5I,L) effects against HG in HUVECs. In the aortic endothelium of db/db mice, we observed that the RES-mediated pro-endothelialization (Supplementary Figures S5A,B) and pro-proliferation (Supplementary Figures S5C,D) effects were diminished after 10058-F4 treatment, while apoptosis was increased (Supplementary Figures S5E,F) in the endothelium. However, systemic 10058-F4 treatment could not reverse RES downregulated fasting blood glucose and plasma insulin levels (Supplementary Figures S1B,C).

**FIGURE 9:**
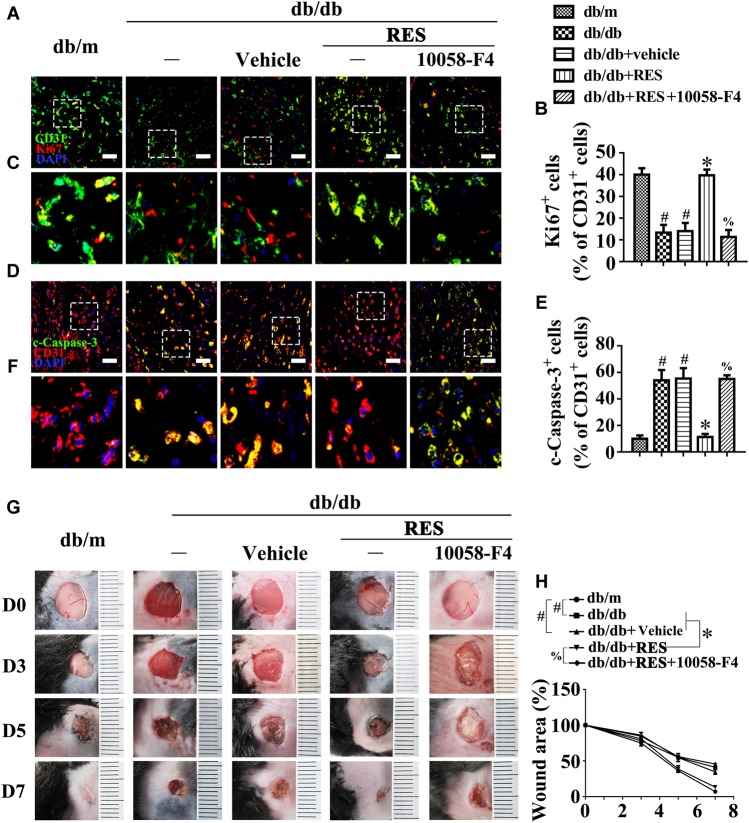
c-Myc participates in the endothelial protective action of RES against hyperglycemia *in vivo*. **(A)** The presence of immunofluorescence with CD31 and Ki67 of wounded skin tissue sections, scale bars = 30 μm (×400), **(C)** enlargement of the indicated area in **(A)**, scale bars = 12 μm (×1000), **(D)** confocal immunofluorescence with CD31 and c-Caspase-3 of wounded skin tissue sections, scale bars = 30 μm (×400), **(F)** enlargement of the indicated area in **(D)**, scale bars = 12 μm (×1000), and **(G)** images of skin wounds, from db/m mice, db/db mice and db/db mice receiving RES (10 μM) or vehicle treatment with saline smeared on the wound, *n* = 6 mice in each group. For signaling pathway analysis, 10058-F4 (50 μM) was injected intradermally into the wound edges in the mice after RES smeared on the wound. Quantification of the proportion of Ki67 positive staining of endothelial cells **(B)**, c-Caspase-3 positive staining of endothelial cells **(E)**, and wound areas **(H)**. All values displayed are means ± SEM of 8 independent experiments. ^#^*p* < 0.05 vs. db/m mice; ^∗^*p* < 0.05 vs. db/db mice or vehicle treated db/db mice; ^%^*p* < 0.05 vs. db/db mice receiving RES.

We then examined the functional role of the RES-mediated increase of c-Myc expression *in vivo* using the skin wound healing model with T2DM mice. Local delivery of 10058-F4 in diabetic mice skin wounds hardly had an influence on the fasting blood glucose and plasma insulin levels (Supplementary Figures S1D,E). But we observed that RES-restored proliferation of endothelial cells along with CD31^+^ capillary density in diabetic regenerative skin tissue was abrogated by 10058-F4 treatment (Figure 9A–C). Meanwhile, RES-inhibited endothelial cell apoptosis was abolished due to 10058-F4 treatment (Figure 9D–F) (Supplementary Figures S5G,H). As a result, RES-accelerated wound healing in db/db mice was also abrogated by 10058-F4 (Figure 9G,H). These results demonstrated that c-Myc, as the main effector of FOXO1, participates in the endothelial protective action of RES against hyperglycemia.

## Discussion

Vascular endothelial cells have important roles in angiogenesis, with substantial contributions to the wound healing process. However, endothelial dysfunction is the earliest and most fundamental pathological change in diabetes and is responsible for diabetic angiopathy. The wound healing process is complex, dynamic, and orderly. The underlying processes of angiogenesis and neovascularization play crucial pathophysiological roles in sustaining newly formed tissues. However, the role of RES in angiogenesis regulation differs. RES inhibits tumor growing through anti-angiogenesis (Kamaleddin, 2016). As for experimental corneal neovascularization, the effect of RES is related to the way of administration, oral RES has anti-angiogenesis effect, while subconjunctival administration has no anti-angiogenesis effect (Doganay et al., 2013). On the contrary, RES promotes random skin flap survival and the ischemic wound healing through effective angiogenesis (Lakshmanan et al., 2019; Lin et al., 2019). Thus, the exact mechanisms of RES and its regulatory roles for angiogenesis, should be analyzed under specific circumstances. Previous studies have demonstrated that RES reduces the size of foot ulcers in subjects with T2DM (Eroğlu et al., 2015; Gokce et al., 2017). Despite the significant protective effect of RES against diabetic non-healing skin ulcers, most studies have not examined the precise signaling molecular mechanisms. Thus the mechanisms mediating these effects have remained uncharacterized and required further analysis. The present study provides novel evidence that RES promotes diabetic wound healing in mouse models of T2DM during hyperglycemia, at least in part, by inhibiting hyperglycemia-triggered endothelial dysfunction, which leads to angiogenic inhibition. This demonstrated that RES activates SIRT1 to participate in diabetic wound healing by inhibiting FOXO1 and then de-repressing the c-Myc signaling pathway.

Acting as a potential agonist of SIRT1, several preclinical studies using animal models have highlighted the beneficial effects of RES on diabetic complications (Yun et al., 2012; Ma et al., 2017). SIRT1 is involved in cell and organismal aging processes, which play crucial roles in regulating senescence and cellular differentiation, and also control metabolic pathways in response to nutrient availability across a broad range of tissues. The beneficial effects of SIRT1 activation in diabetic vasculopathy have been demonstrated in previous studies (Zhou et al., 2011; Hammer et al., 2017). The SIRT1 activation by RES improves palmitate-induced inflammation and insulin resistance, ameliorates the HG-induced impairment of HUVECs, induces mitochondrial biogenesis in aortic endothelial cells of db/db mice, and improves aortic dysfunction in diabetic mice (Csiszar et al., 2009; Zhou et al., 2015; Liu et al., 2016). The present study demonstrates the expanded role of SIRT1 as a key regulator of endothelial cell homeostasis and identifies SIRT1 as a specific modulator of the angiogenic activity of endothelial cells against hyperglycemia. To dissect the roles of SIRT1 in the endothelial protective action and angiogenic activity of RES against HG, we used *SIRT1* siRNA to interfere with SIRT1 expression in HUVECs. In the absence of *SIRT1*, the RES-modulated angiogenic activity of endothelial cells was largely attenuated. This result was further confirmed by the SIRT1 inhibitor EX-527 in aortic endothelial cells and diabetic wounds from db/db mice.

Silent information regulator 1 interacts with a series of substrates, such as FOXOs, p53, nuclear factor-κB, peroxisome proliferator-activated receptor-γ coactivator-1α, and myoblast determination protein, which mediate the specific functions of SIRT1 (Feige and Auwerx, 2008). The interaction between SIRT1 and FOXOs was documented previously during angiogenic signaling (Potente et al., 2007). FOXO1 is a member of the FOXO transcription factor family and is highly expressed in the vascular endothelium, but its role in modulating endothelial function differs under various circumstances. Global deletion of FOXO1 during embryonic stage leads to embryonic lethality due to severe vascular defects (Furuyama et al., 2004), suggesting an important role of FOXO1 in maintaining vascular development. But in type 1 and type 2 diabetic rats, high glucose promotes FOXO1 nuclear translocation, leading to apoptosis initiation and loss of rat microvascular endothelial cells (Behl et al., 2009), suggesting that FoxO1 is an essential negative transcriptional regulator of vessel formation. Consistent with this view, we demonstrated in the present study that the level of FOXO1 protein was dramatically increased by hyperglycemia, while it was decreased by RES against hyperglycemia. To clarify the roles of FOXO1 in the endothelial protective and proangiogenic action of RES against HG, we overexpressed FOXO1 with adenovirus in HUVECs and diabetic wounds from db/db mice, and FOXO1 overexpression largely attenuated the RES-modulated angiogenic activity of endothelial cells.

The biological function of FOXO1 closely correlated with its nuclear localization and subsequent transcription activity. SIRT1-mediated deacetylation has been recognized as a well-known manner to regulate FOXO1 activity, SIRT1 controls the nuclear shuttling of FOXOs and regulates their activity either positively or negatively depending on the target gene or cell type (Giannakou and Partridge, 2004). In tamoxifen-resistant MCF-7 breast cancer cells, SIRT1 inhibition reduces nuclear FOXO1 levels and the expression of downstream resistance protein 2 (Choi et al., 2013). However, in insulin-resistant 3T3-L1 adipocytes, SIRT1 enhances the shuttling of FOXO1 from the nucleus to the cytoplasm where protein degradation occurs (Chen et al., 2018). In the present study, we found that HG-mediated SIRT1 inhibition increased nuclear FOXO1 levels, while RES-mediated SIRT1 activation facilitated FOXO1 translocation from the nucleus to the cytoplasm. Thus, the exact mechanisms of the SIRT1/FOXO1 pathway and its regulatory roles in endothelial dysfunction and angiogenesis should be analyzed under specific circumstances.

Members of the FOXO family participate in the regulation of *c-Myc* gene expression (Masui et al., 2013). FOXO1 overexpression suppresses c-Myc expression, whereas *FOXO1* depletion enhances c-Myc levels in HUVECs, and endothelial cells derived from mutant mice (Wilhelm et al., 2016). In this study, we observed that increased FOXO1 expression in HUVECs exposed to HG led to the inhibition of c-Myc expression as a process of cellular damage in endothelial cells, while RES-mediated SIRT1 activation promoted the degradation of FOXO1, which resulted in the de-repression of c-Myc expression under HG. *c-Myc* is the most extensively studied *Myc* gene with key regulatory roles in a wide array of cellular processes including the regulation of cell cycle progression, cell proliferation, differentiation, transformation, angiogenesis, and apoptosis. *c-Myc* ablation impairs glycolysis, mitochondrial function, and proliferation of endothelial cells, while its endothelial cell-specific overexpression fuels these processes (Baudino et al., 2002). To verify the roles of c-Myc in the endothelial protective action and angiogenic activity of RES against HG, we used si-*c-Myc* to interfere with its expression in HUVECs. In the absence of *c-Myc*, the RES-modulated angiogenic activity of endothelial cells against HG was largely attenuated. This result was further confirmed by the treatment of aortic endothelial cells and diabetic wounds from db/db mice with the c-Myc inhibitor 10058-F4.

In addition, we realized that there were some limitations in the present study. We utilized mouse skin wound healing model to evaluate the endothelial protective role of RES under diabetes conditions. However, the mouse dermal wounds heal largely by contraction whereas human dermal wounds heal largely by the production of new tissue. Although we made no attempt to limit contraction in the murine dermal wounds, we have verified a proangiogenic role of RES in diabetic mouse skin wound healing model, which could dramatically promote endothelial cells proliferation, and neovascularization. The proangiogenic property of RES might provide basis for its application in human wounds healing.

**FIGURE 10:**
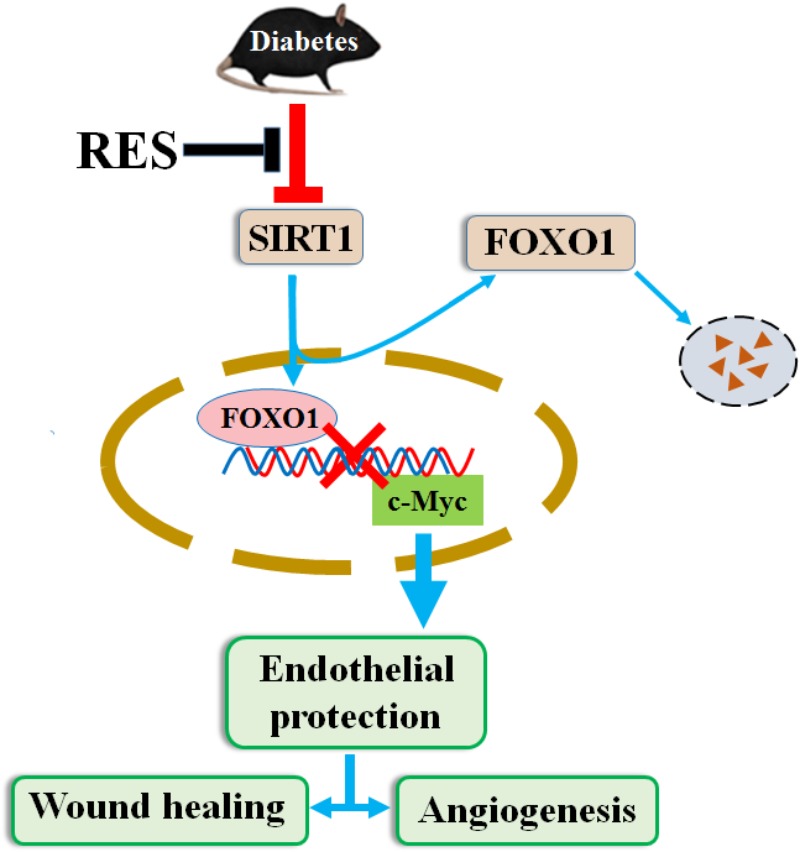
RES promotes diabetic wound healing via SIRT1-FOXO1-c-Myc signaling pathway mediated angiogenesis. Schematic illustration of the protective effects of RES on endothelial cells under HG conditions. HG decreases expression of SIRT1 in endothelial cells and induces endothelial dysfunction, which impairs angiogenic function of endothelial cells. Under HG conditions co-treatment with RES improves endothelial cells survival and function, which relies on RES as agonist of SIRT1, stimulates c-Myc expression through promoting FOXO1 degradation.

In summary, our results have uncovered a novel pathway by which RES prevents hyperglycemia-induced endothelial dysfunction and angiogenic impairment. This pathway, which relies on RES as an agonist of SIRT1, stimulates c-Myc expression by promoting FOXO1 degradation under HG (Figure 10). The results from this study may have implications for the pathogenesis and treatment of diabetic non-healing skin ulcers and other diabetes-associated vascular complications.

## Conclusion

Resveratrol accelerated diabetic wound healing via its endothelial protective and proangiogenic effects. Mechanistically, RES activated endothelial SIRT1 and promoted FOXO1 degradation, which further de-repressed c-Myc expression against HG.

## Ethics Statement

All animal experiments and methods performed in this study followed ethical guidelines for animal studies, and were approved by the Institutional Animal Care and Use Committee of Wenzhou Medical University after obtaining their ethical approval to pursue this study (wydw2017-0026).

## Author Contributions

XH, JiaS, and GC conceived the study, acquired the data, interpreted the results, and drafted the manuscript. CN, YW, CZ, and JianS performed the some cell experiments. HH, SH, YL, and YS assisted the technicians with animal sacrifice. WC, LJ, and ZZ designed the study, interpreted the results, and revised the manuscript. All authors read and approved the final manuscript.

## Conflict of Interest Statement

The authors declare that the research was conducted in the absence of any commercial or financial relationships that could be construed as a potential conflict of interest.
