# Supplementary material for: Resveratrol Promotes Diabetic Wound Healing via SIRT1-FOXO1-c-Myc Signaling Pathway-Mediated Angiogenesis
Source: Front Pharmacol. 2019 Apr 24;10:421. doi: 10.3389/fphar.2019.00421 (PMC6491521; doi:10.3389/fphar.2019.00421)
Supplement: Supplementary file 1 [file Data_Sheet_1.doc]

**Supplemental Material**

**to**

**Resveratrol Promotes Diabetic Wound Healing via SIRT1-FOXO1-c-Myc Signaling Pathway-mediated Angiogenesis**

Xiaozhong Huang^1, 2†^, Jia Sun^2†^, Gen Chen^2†^, Chao Niu^3^, Ying Wang^4^, Congcong Zhao^2^, Jian Sun^2^, Huiya Huang^5^, Shuai Huang^2^, Yangzhi Liang^2^, Yingjie Shen^2^, Weitao Cong^2^, Litai Jin^2*^, Zhongxin Zhu^2*^

**Fig. S1:** Immunohistochemical staining of negative antibody control for skin tissue (scale bars=30 μm (×400)), endothelial cells (scale bars=100 μm (×200)), and aorta with different magnification (scale bars=200 μm (×40), scale bars=20 μm (×400), respectively) (**A**). Quantification of fasting blood glucose (**B**) and plasma insulin levels (**C**), from db/m mice, db/db mice and db/db mice receiving RES (50 mg/kg/day) or vehicle treatment with saline infusion, n=6 mice in each group. For signaling pathway analysis, EX-527 inhibitor of SIRT1, was administered at the dose of 5 mg/kg/day, 10058-F4 inhibitor of c-Myc, was administered at the dose of 30 mg/kg/day. Quantification of fasting blood glucose (**D**) and plasma insulin levels (**E**), from db/m mice, db/db mice and db/db mice receiving RES (10 μM) or vehicle treatment with saline smeared on the wound, n=6 mice in each group. For signaling pathway analysis, Ad-FOXO1 was injected intradermally into the wound edges in the mice the day before wounding, EX-527 (10 μM) and 10058-F4 (50 μM) was injected intradermally into the wound edges in the mice after RES smeared on the wound. n=6 mice in each group. All values displayed are means ± SEM of 8 independent experiments. ^ p < 0.05 vs. db/m mice; $ p < 0.05 vs. db/db mice or vehicle treated db/db mice.

**Fig. S2: RES attenuates hyperglycemia-induced endothelial dysfunction**. The presence of immunofluorescence with (**A**) CD31, scale bars=200 μm (×40) and (**C**) PCNA, scale bars=20 μm (×400), (**E**) endothelial cells TUNEL assay, scale bars=20 μm (×400), from db/m mice, db/db mice and db/db mice receiving RES (50 mg/kg/day) or vehicle treatment with saline infusion aorta tissue sections, n=6 mice in each group. Quantification of the CD31 positive area (**B**), the proportion of PCNA-positive cells (**D**), the proportion of TUNEL-positive cells (**F**). (**G**) Confocal immunofluorescence with CD31 of wounded skin tissue sections, scale bars=30 μm (×400), db/db mice and db/db mice receiving RES (10 μM) or vehicle treatment with saline smeared on the wound, n=6 mice in each group. (**H**) Quantification of the proportion of CD31-positive cells in (G). (**I**) The presence of aortic rings from C57BL/6 mice, scale bars=500 μm (×20), cultured in different mediums containing NG (5.5 mM), HG (33 mM) alone or with RES (10 μM) for 72 h, mannitol (MAN; 33 mM: 5.5 mM of glucose + 27.5 mM of _D_-mannitol) was served as the osmotic control for the HG. (**J**) Quantification of the number of sprouts. (**K**) Cell lysates of HUVECs were used to detect the Bax, Bcl-2, c-Caspase-3 as well as PCNA protein levels by immunoblotting. HUVECs treated as indicated in (I). (**L, M, N**) The quantitative analysis of each immunoblots. All values displayed are means ± SEM of 8 independent experiments. ^ p < 0.05 vs. db/m mice; $ p < 0.05 vs. db/db mice or vehicle treated db/db mice. # p < 0.05 vs. NG or MAN; * p < 0.05 vs. HG.

**Fig. S3: The endothelial protective action of RES against HG is SIRT1 dependent**. The presence of immunofluorescence with (**A**) CD31, scale bars=200 μm (×40) and (**C**) PCNA, scale bars=20 μm (×400), (**E**) endothelial cells TUNEL assay, scale bars=20 μm (×400), from db/m mice, db/db mice and db/db mice receiving RES (50 mg/kg/day) or vehicle treatment with saline infusion aorta tissue sections, n=6 mice in each group. For signaling pathway analysis, EX-527 inhibitor of SIRT1, was administered at the dose of 5 mg/kg/day. Quantification of the CD31 positive area (**B**), the proportion of PCNA-positive cells (**D**), the proportion of TUNEL-positive cells (**F**). (**G**) Confocal immunofluorescence with CD31 of wounded skin tissue sections, scale bars=30 μm (×400), from db/m mice, db/db mice and db/db mice receiving RES (10 μM) or vehicle treatment with saline smeared on the wound, n=6 mice in each group. For signaling pathway analysis, EX-527 (10 μM) was injected intradermally into the wound edges in the mice after RES smeared on the wound. (**H**) Quantification of the proportion of CD31-positive cells in (G). Cell lysates of HUVECs were used to detect (**I**) the Bax, Bcl-2, c-Caspase-3 as well as PCNA protein levels by immunoblotting. HUVECs were cultured either in NG or HG medium in the presence or absence of RES (10 μM) for 72 h, MAN was served as the osmotic control for the HG. For signaling pathway analysis, EX-527 (10 μM) was pretreated for 2 h before RES administration. (**J, K, L**) The quantitative analysis of each immunoblots. (**M, N, O**) The mRNA and protein expression levels of SIRT1 in the aortic homogenates form *SIRT1* flox/flox; *Tie2-Cre* (+) mice and its control littermates *SIRT1* flox/flox; *Tie2-Cre* (-). (**P**) Representative confocal images of aortic rings from *SIRT1* flox/flox; *Tie2-Cre* (+) mice and its control littermates *SIRT1* flox/flox; *Tie2-Cre* (-) mice cultured in different mediums containing NG, HG; alone or with RES (10 μM) for 72 h, Scale bars=500 μm (×20). (**Q**) Quantification of the number of sprouts. All values displayed are means ± SEM of 8 independent experiments. ^ p < 0.05 vs. db/m mice; $ p < 0.05 vs. db/db mice or vehicle treated db/db mice; & p < 0.05 vs. db/db mice receiving RES; # p < 0.05 vs. NG or MAN; * p < 0.05 vs. HG; % p < 0.05 vs. HG co-incubated with RES; △ p < 0.05 vs. *SIRT1* flox/flox; *Tie2-Cre* (-) mice.

**Fig. S4: FOXO1 participates in the endothelial protective action of RES against hyperglycemia**. (**A**) Confocal immunofluorescence with CD31 of wounded skin tissue sections, scale bars=30 μm (×400), from db/m mice, db/db mice and db/db mice receiving RES (10 μM) or vehicle treatment with saline smeared on the wound, n=6 mice in each group. For signaling pathway analysis, Ad-*FOXO1* was injected intradermally into the wound edges in the mice the day before wounding. (**B**) Quantification of the proportion of CD31-positive cells in (A). (**C**) Cell lysates of HUVECs were used to detect the Bax, Bcl-2, c-Caspase-3 as well as PCNA protein levels by immunoblotting. (**D, E, F**) The quantitative analysis of each immunoblots, (**G, I**) OCR and (**H, J**) cellular ATP. All values displayed are means ± SEM of 8 independent experiments. # p < 0.05 vs. NG or MAN; * p < 0.05 vs. HG; % p < 0.05 vs. HG co-incubated with RES. ^ p < 0.05 vs. db/m mice; $ p < 0.05 vs. db/db mice or vehicle treated db/db mice; & p < 0.05 vs. db/db mice receiving RES.

**Fig. S5: c-Myc participates in the endothelial protective action of RES against hyperglycemia**. The presence of immunofluorescence with (**A**) CD31, scale bars=200 μm (×40) and (**C**) PCNA, scale bars=20 μm (×400), (**E**) endothelial cells TUNEL assay, scale bars=20 μm (×400), from db/m mice, db/db mice and db/db mice receiving RES (50 mg/kg/day) or vehicle treatment with saline infusion aorta tissue sections, n=6 mice in each group. For signaling pathway analysis, 10058-F4 inhibitor of c-Myc, was administered at the dose of 30 mg/kg/day. Quantification of the CD31 positive area (**B**), the proportion of PCNA-positive cells (**D**), the proportion of TUNEL-positive cells (**F**). (**G**) Confocal immunofluorescence with CD31 of wounded skin tissue sections, scale bars=30 μm (×400), db/db mice and db/db mice receiving RES (10 μM) or vehicle treatment with saline smeared on the wound, n=6 mice in each group. For signaling pathway analysis, 10058-F4 (50 μM) was injected intradermally into the wound edges in the mice after RES smeared on the wound. (**H**) Quantification of the proportion of CD31-positive cells in (G). (**I**) Cell lysates of HUVECs were used to detect the Bax, Bcl-2, c-Caspase-3 as well as PCNA protein levels by immunoblotting. HUVECs were cultured either in NG or HG medium in the presence or absence of RES (10 μM) for 72 h. For signaling pathway analysis, 10058-F4 (50 μM) was pretreated for 2 h before RES administration. (**J, K, L**) The quantitative analysis of each immunoblots. All values displayed are means ± SEM of 8 independent experiments. # p < 0.05 vs. NG or MAN; * p < 0.05 vs. HG; % p < 0.05 vs. HG co-incubated with RES. ^ p < 0.05 vs. db/m mice; $ p < 0.05 vs. db/db mice or vehicle treated db/db mice; & p < 0.05 vs. db/db mice receiving RES.


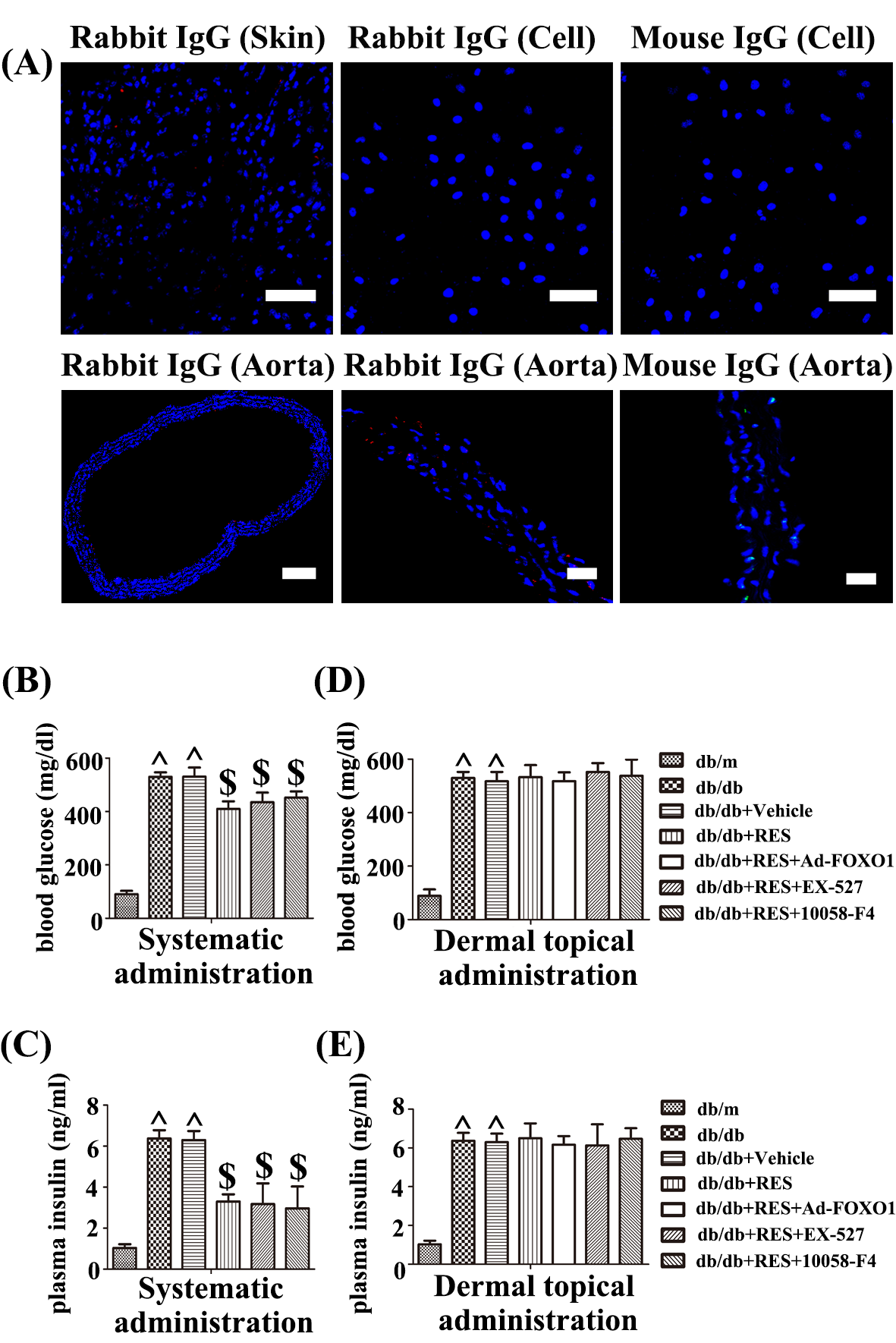


Figure S1


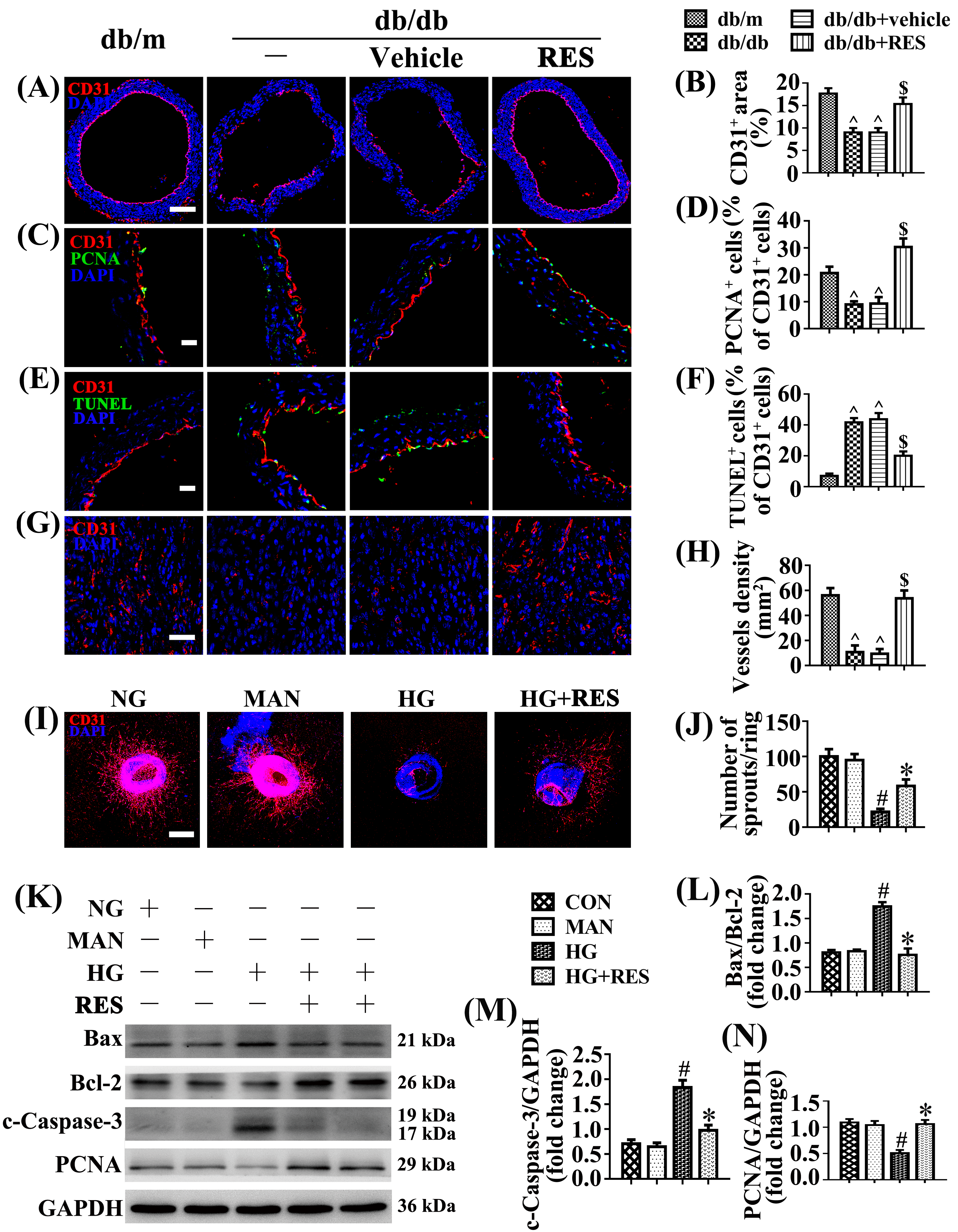


Figure S2


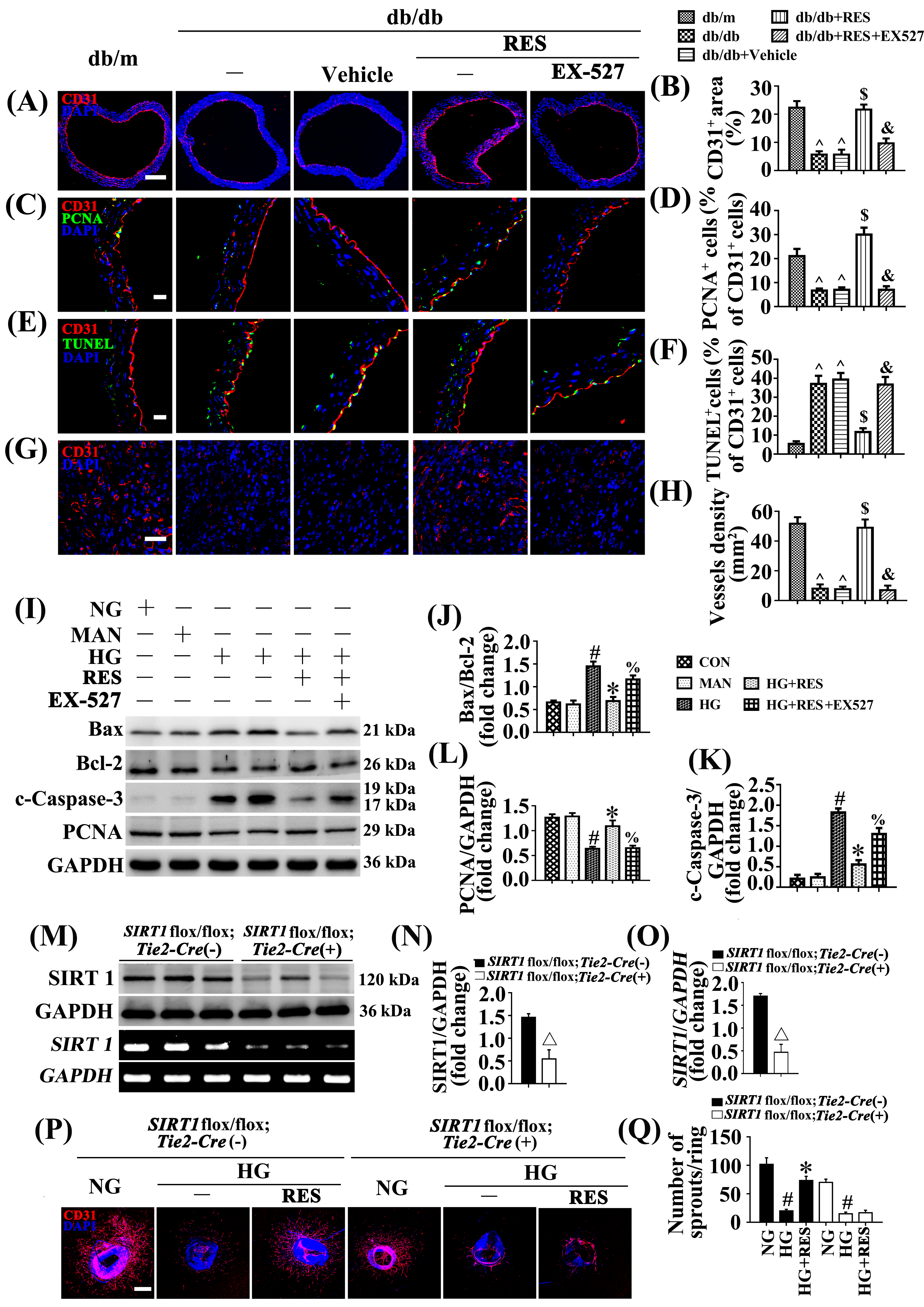


Figure S3


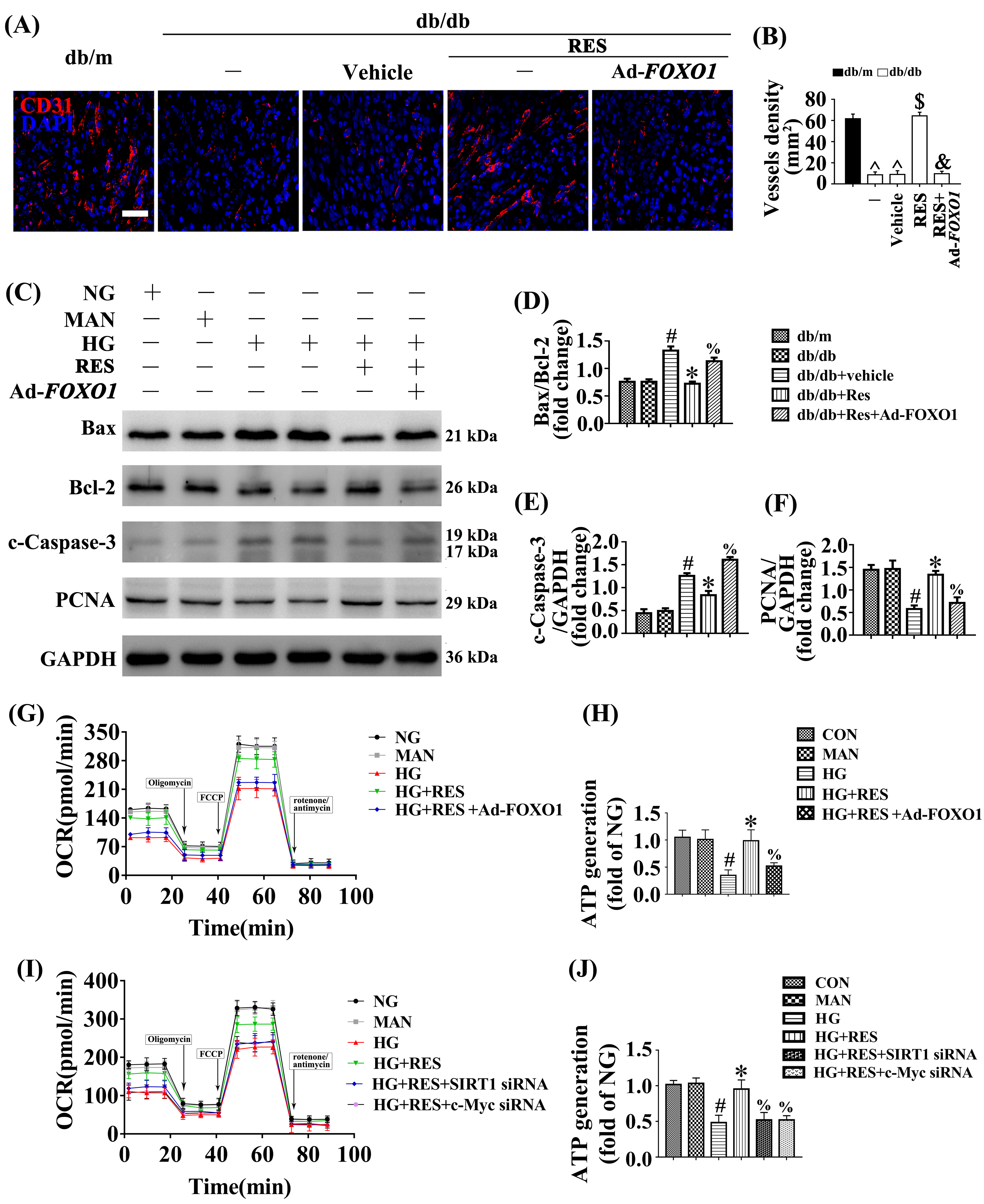


Figure S4


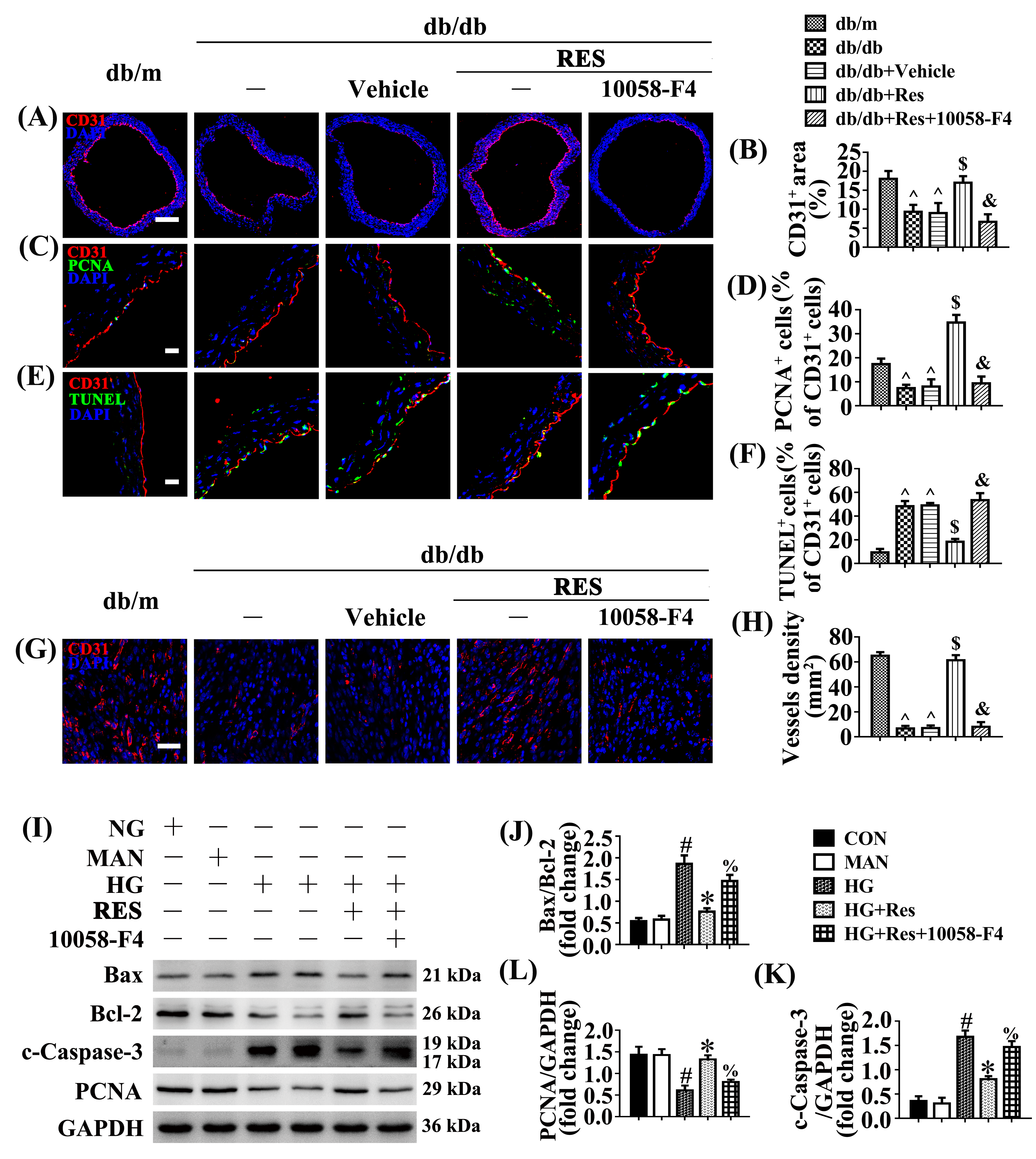


Figure S5
